# Biosynthesis of 3,6-Dideoxy-heptoses for the Capsular Polysaccharides of *Campylobacter jejuni*

**DOI:** 10.1021/acs.biochem.3c00012

**Published:** 2023-03-21

**Authors:** Manas K. Ghosh, Dao Feng Xiang, Frank M. Raushel

**Affiliations:** Department of Chemistry, Texas A&M University, College Station, Texas 77845, United States

## Abstract

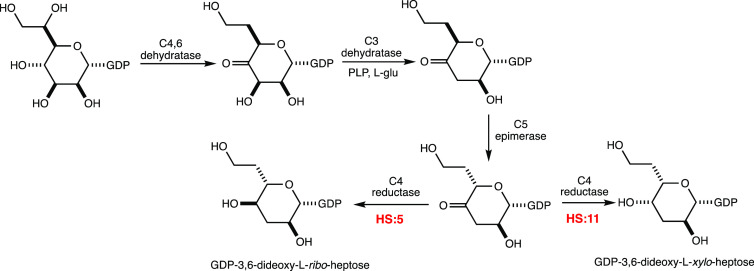

*Campylobacter jejuni* is the leading cause of food poisoning in the United States. Surrounding the exterior surface of this bacterium is a capsular polysaccharide (CPS) that helps protect the organism from the host immune system. The CPS is composed of a repeating sequence of common and unusual sugar residues, including relatively rare heptoses. In the HS:5 serotype, we identified four enzymes required for the biosynthesis of GDP-3,6-dideoxy-β-l-*ribo*-heptose. In the first step, GDP-d-*glycero*-α-d-*manno*-heptose is dehydrated to form GDP-6-deoxy-4-keto-α-d-*lyxo*-heptose. This product is then dehydrated by a pyridoxal phosphate-dependent C3-dehydratase to form GDP-3,6-dideoxy-4-keto-α-d-*threo*-heptose before being epimerized at C5 to generate GDP-3,6-dideoxy-4-keto-β-l-*erythro*-heptose. In the final step, a C4-reductase uses NADPH to convert this product to GDP-3,6-dideoxy-β-l-*ribo*-heptose. These results are at variance with the previous report of 3,6-dideoxy-d-*ribo*-heptose in the CPS from serotype HS:5 of *C. jejuni*. We also demonstrated that GDP-3,6-dideoxy-β-l-*xylo*-heptose is formed using the corresponding enzymes found in the gene cluster from serotype HS:11 of *C. jejuni*. The utilization of different C4-reductases from other serotypes of *C. jejuni* enabled the formation of GDP-3,6-dideoxy-α-d-*arabino*-heptose and GDP-3,6-dideoxy-α-d-*lyxo*-heptose.

## Introduction

**Figure 1:**
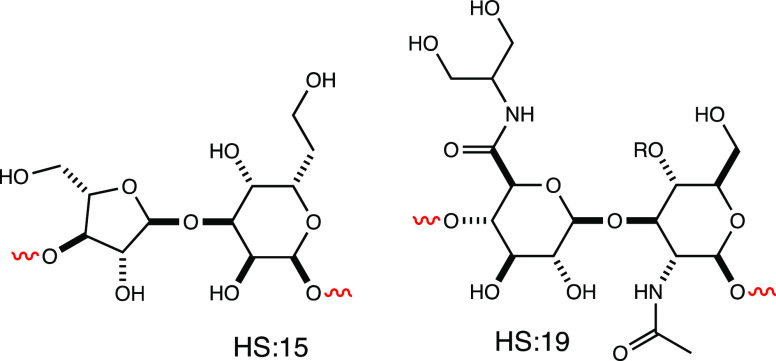
Structures of the repeating sugars in the CPSs from serotypes HS:15^4^ and HS:19^5^ of *C. jejuni*. The CPS from the HS:15 serotype contains l-arabinose and 6-deoxy-l-*gulo*-heptose, whereas the CPS from the HS:19 serotype contains the serinol amide of d-glucuronate and *N*-acetyl d-glucosamine. The *N*-acetyl d-glucosamine moiety from the CPS from HS:19 may also be modified nonstoichiometrically at C4 with a methyl phosphoramidate group.

The exterior surface of the human pathogen *Campylobacter jejuni* is coated with a capsular polysaccharide (CPS) that helps protect the organism from the host immune system.^1^ The CPS is anchored to the cell wall and is composed of a repeating polysaccharide sequence that can be further decorated by methylation, methyl phosphoramidylation, amidation, and other chemical modifications.^2,3^ With *C. jejuni*, at least 33 different strains or serotypes have been identified, and the gene clusters required for the expression of the genes needed for the assembly of the CPS have been sequenced.^2^ In addition, the chemical structures of the repeating polysaccharides have been determined for at least 12 of the known serotypes.^2^ Among the most common monosaccharides that have been identified thus far in the CPS of *C. jejuni* are the relatively rare seven-carbon heptoses. To date, 10 structurally distinct heptoses have been chemically identified. The repeating polysaccharides identified from the HS:15 and HS:19 serotypes are presented in Figure 1.

**Figure 2:**
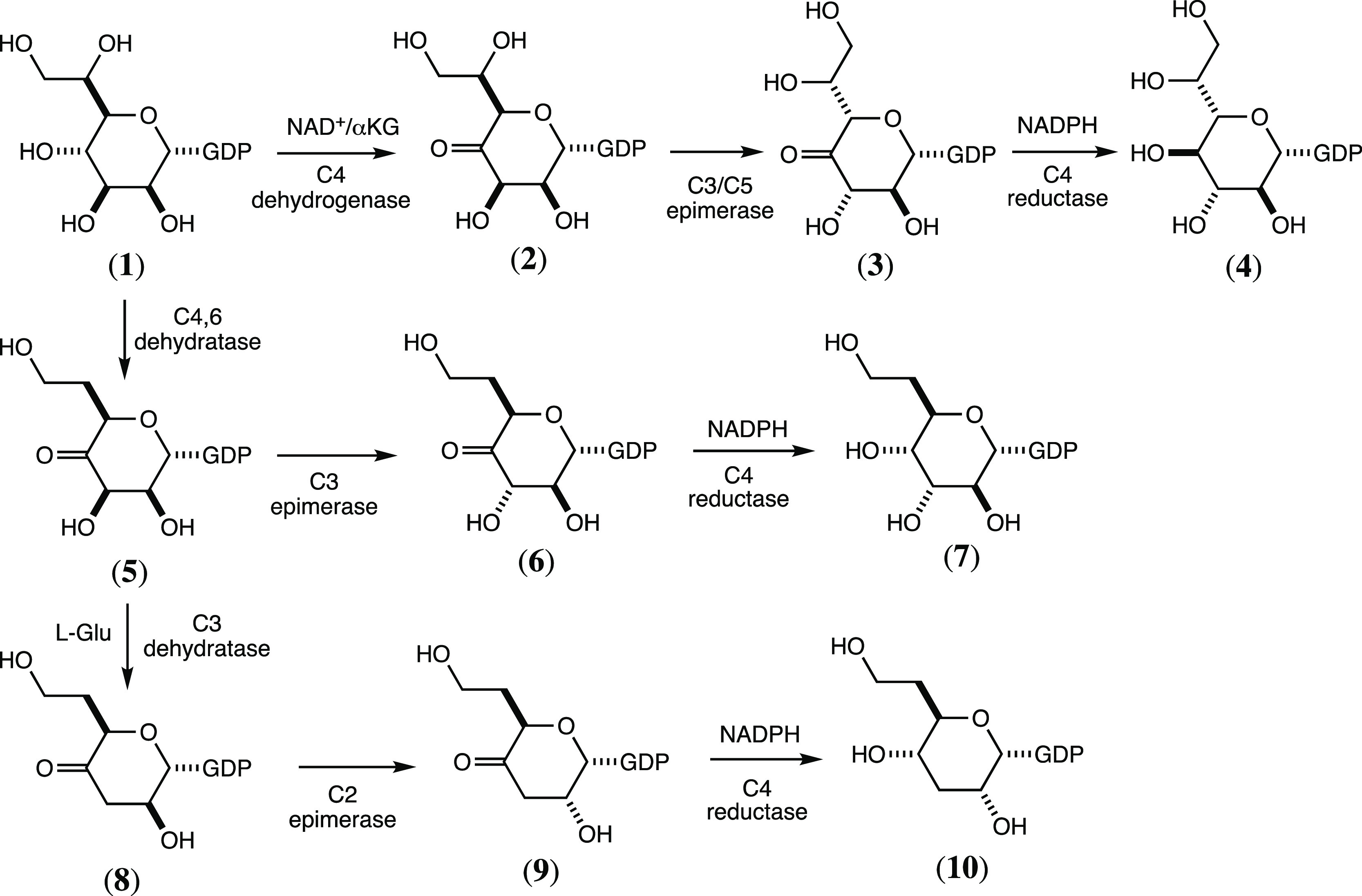
Biosynthetic pathways for the formation of GDP-d-*glycero*-β-l-*gluco*-heptose (**4**) and GDP-6-deoxy-α-d-*altro*-heptose (**7**) and the initially proposed pathway for the biosynthesis of GDP-3,6-dideoxy-α-d-*ribo*-heptose (**10**).

All of the currently identified heptoses found in the CPS of *C. jejuni* are thought to arise from the modification of GDP-d-*glycero*-α-d-*manno*-heptose (**1**).^6−15^ For example, in the HS:2 serotype, we have shown that this compound is transformed into GDP-d-*glycero*-β-l-*gluco*-heptose (**4**) by the sequential action of a C4-dehydrogenase, a C3/C5-isomerase, and a C4-reductase via the formation of intermediates **2** and **3**, as illustrated in Figure 2.^16^ We and the Creuzenet group have shown that 6-deoxy-heptoses are also formed from GDP-d-*glycero*-α-d-*manno*-heptose (**1**) through the catalytic activities of a C4,6-dehydratase, a C3- or C3/C5-isomerase, and a C4-reductase.^9−11,14,15^ The biosynthesis of GDP-6-deoxy-α-d-*altro*-heptose (**7**) from serotype HS:23/36 is shown in Figure 2 via the intermediacy of compounds **5** and **6.**(9−11,14) Recently, we have identified biosynthetic pathways for the formation of five additional 6-deoxy-heptoses in various serotypes and strains of *C. jejuni*.^14^ These heptoses include GDP-6-deoxy-α-d-*ido*-heptose, GDP-6-deoxy-α-d-*manno*-heptose, GDP-6-deoxy-β-l-*gulo*-heptose, GDP-6-deoxy-β-l-*galacto*-heptose, and GDP-6-deoxy-β-l-*gluco*-heptose.^14^

**Figure 3:**
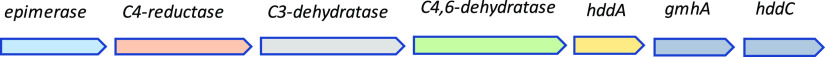
Portion of the gene cluster from the HS:5 serotype of *C. jejuni* that is required for the biosynthesis of the 3,6-dideoxy-heptose moiety of the CPS. The individual genes are not drawn to the appropriate relative length.

The least common heptose currently identified in the CPS of *C. jejuni* is 3,6-dideoxy-d-*ribo*-heptose (**10**) reported in the HS:5 serotype.^2,17,18^ A portion of the gene cluster for the biosynthesis of the CPS of the HS:5 serotype is shown in Figure 3. Here, one can clearly identify the three genes needed for the biosynthesis of GDP-d-*glycero*-α-d-*manno*-heptose. These include *hddC* for the expression of d-*glycero*-α-d-*mann*o-heptose 1-phosphate guanosyl transferase; *gmhA* for phosphoheptose isomerase; and *hddA* for the production of d-heptose-7-phosphate kinase. The proposed pathway for the synthesis of GDP-3,6-dideoxy-α-d-*ribo*-heptose (**10**) is shown in Figure 2. In this proposed pathway, GDP-d-*manno*-heptose-4,6-dehydratase converts GDP-d-*glycero*-α-d-*manno*-heptose (**1**) into GDP-6-deoxy-4-keto-α-d-*lyxo*-heptose (**5**). In the next step, a C3-dehydratase catalyzes the pyridoxal phosphate (PLP)-dependent removal of the hydroxyl group at C3 to form GDP-3,6-dideoxy-4-keto-α-d-*threo*-heptose (**8**), which is followed by the epimerization of C2 to form GDP-3,6-dideoxy-4-keto-α-d-*erythro*-heptose (**9**). In the final step, the C4-reductase reduces the carbonyl group at C4 to form GDP-3,6-dideoxy-α-d-*ribo*-heptose (**10**). A nearly identical set of seven genes is found within the gene cluster for the CPS from serotype HS:45. However, within the gene cluster for serotype HS:11, the same set of genes is found except for the putative C4-reductase where the sequence identity to the C4-reductase from HS:5 is only 41%, suggesting that the stereochemistry will be different at C4, relative to GDP-3,6-dideoxy-α-d-*ribo*-heptose (**10**).

Here, we have functionally characterized the four enzymes that are apparently needed for the biosynthesis of 3,6-dideoxy-heptoses in *C. jejuni* from the HS:5 and HS:11 serotypes. Contrary to expectations, the four enzymes from serotype HS:5 ultimately make GDP-3,6-dideoxy-β-l-*ribo*-heptose (**12**) rather than the expected GDP-3,6-dideoxy-α-d-*ribo*-heptose (**10**). The putative epimerase was shown to catalyze the epimerization of C5 rather than the anticipated epimerization of C2. We have also shown that the enzymes from serotype HS:11 make GDP-3,6-dideoxy-β-l-*xylo*-heptose (**13**).

## Materials and Methods

### Materials

Lysogeny broth (LB) medium, isopropyl-β-d-thiogalactopyranoside (IPTG), and NADPH were purchased from Research Products International. The protease inhibitor cocktail, lysozyme, DNase I, glutamate dehydrogenase, acetaldehyde dehydrogenase, acetaldehyde, kanamycin, imidazole, Tris, HEPES, PLP, and l-glutamate were obtained from Sigma-Aldrich. Ammonium bicarbonate, potassium phosphate, 2-mercaptoethanol, KCl, and MgCl_2_ were also acquired from Sigma-Aldrich. DNase I was purchased from Roche. Vivaspin 20 spin filters and HisTrap and HiTrap Q columns were obtained from Cytiva. The 10 kDa Nanosep spin filters were purchased from Pall Corporation (Port Washington, NY). Deuterium oxide was acquired from Cambridge Isotope Laboratories Inc., and oxygen-18 labeled water (98%) was obtained from Medical Isotopes Inc.

### Equipment

Ultraviolet spectra were collected on a SpectraMax 340 (Molecular Devices) ultraviolet–visible plate reader using 96-well Greiner plates. ^1^H NMR spectra were recorded on a Bruker Avance III 400 MHz system equipped with a broadband probe and sample changer. Mass spectrometry data were collected on a Thermo Scientific Q Exactive Focus system run in negative ion mode.

### Plasmid Construction

The DNA constructs for the expression of the genes for the C3-dehydratases, epimerases, and C4-reductases from *C. jejuni* serotypes HS:5 and HS:11 were chemically synthesized and codon-optimized by Twist Biosciences (San Francisco, CA). The genes included the C3-dehydratases from HS:5 (UniProt entry: A0A0U3ALB0) and HS:11 (UniProt entry: A0A0U2RG51); the epimerases from HS:5 (UniProt entry: A0A0Q3UFL0) and HS:11 (UniProt entry: A0A0U2QGV6); and the C4-reductases from HS:5 (UniProt entry: A0A0U3C2F2) and HS:11 (UniProt entry: A0A0U3ANW2). The DNA was inserted between the NdeI and XhoI restriction sites of a pET-28a (+) expression vector. These constructs encode for the expression of an N-terminal His_6_-affinity tag, and the complete amino acid sequences of the proteins purified for this investigation are shown in Figure S1.

### Protein Expression and Purification

The C3-dehydratases, epimerases, and C4-reductases from the HS:5 and HS:11 serotypes were purified according to the procedures reported previously.^12−15^*Escherichia coli* BL21(DE3) competent cells were transformed by the appropriate plasmids. Single colonies were inoculated in 50 mL of LB medium (20 g/L of yeast extract, 35 g/L of tryptone, 5 g/L of sodium chloride, pH 7.0) supplemented with 50 μg/mL of kanamycin and grown at 37 °C overnight with shaking. The starter cultures were used to inoculate 1 L of LB medium, grown at 37 °C with shaking to an OD_600_ of ∼0.8. Expression was induced by the addition of IPTG to a final concentration of 1.0 mM. The cultures were subsequently incubated for 18 h at 15 °C with shaking at 140 rpm. The cells were harvested by centrifugation at 7000 × *g* for 10 min at 4 °C, frozen in liquid N_2_, and stored at −80 °C.

Purification of the two C3-dehydratases, C5-epimerases, and C4-reductases from serotypes HS:5 and HS:11 were conducted at 22 °C. In a typical purification, ∼5 g of frozen cell paste was resuspended in 50 mL of buffer A (50 mM HEPES, pH 7.5, 250 mM KCl, 5.0 mM imidazole) supplemented with 0.1 mg/mL of lysozyme, 0.05 mg/mL of protease inhibitor cocktail powder, 40 U/mL of DNase I, and 10 mM MgCl_2_. The suspended cells were lysed by sonication (Branson 450 Sonifier), and the supernatant solution was collected after centrifugation at 10,000 × *g* for 30 min. The supernatant solution was loaded onto a prepacked 5 mL HisTrap column and eluted with a linear gradient of buffer B (50 mM HEPES, pH 7.5, 250 mM KCl, 500 mM imidazole). Fractions containing the desired protein, as identified by sodium dodecyl sulfate–polyacrylamide gel electrophoresis, were combined and concentrated in a 20 mL spin filter with a 10 kDa molecular weight cutoff. The imidazole was removed from the protein by dialysis using buffer C (50 mM HEPES, pH 7.5, 250 mM KCl). The protein was concentrated to 5–10 mg/mL, aliquoted, frozen in liquid N_2_, and stored at −80 °C. Typical yields of 10–15 mg for the two C3-dehydratases, 20–30 mg for the two epimerases, and 10–15 mg for the two C4-reductases were obtained from ∼1 L of cell culture.

### Determination of Protein Concentrations

Concentrations of the proteins were determined spectrophotometrically using computationally derived molar absorption coefficients at 280 nm.^19^ The values of ε_280_ (M^–1^ cm^–1^) used for the two C3-dehydratases, C5-epimerases, and C4-reductases from serotypes HS:5 and HS:11 were 51,340, 49,390, 38,850, 51,340, 47,900, and 43,922, respectively.

### Isolation of the C3-Dehydratase Product

The reactions were conducted at 22 °C in either H_2_O or D_2_O at pH 7.5 (or pD 7.5). A 1.0 mL reaction mixture containing 4.0 mM GDP-d-*glycero*-α-d-*manno*-heptose (**1**), 0.2 mM PLP, and 8.0 mM l-glutamate was incubated with GDP-α-d-*manno*-heptose 4,6-dehydratase (4.0 μM) and C3-dehydratase (4.0 μM) in 50 mM phosphate/KOH and 50 mM KCl for 18 h. The reaction was terminated by removing the enzyme from the reaction mixture using a 0.5 mL spin filter with a 10 kDa molecular weight cutoff. The resulting flow-through was injected onto a BioRad FPLC system equipped with a 1.0 mL HiTrap Q HP column. The column was washed with water, and the product was eluted using a linear gradient (0–60%) of 500 mM NH_4_HCO_3_, pH 8.0, over 60 column volumes. Fractions of 0.5 mL were collected and subsequently lyophilized to dryness under vacuum. The resulting samples were reconstituted in either D_2_O or H_2_O and analyzed by ^1^H NMR spectroscopy and mass spectrometry. All ^1^H NMR and two-dimensional ^1^H-^1^H COSY spectra were recorded on a Bruker Avance III 400 MHz NMR spectrometer at 22 °C. Electrospray ionization mass spectrometry (ESI-MS) experiments were performed using a Thermo Scientific Q Exactive Focus.

### Isolation of the C4-Reductase Product

The reactions were conducted at 22 °C in either H_2_O or D_2_O at pH 7.5 (or pD 7.5). A 1.0 mL reaction containing 4.0 mM GDP-3,6-dideoxy-4-keto-α-d-*threo*-heptose (**8**), 0.15 mM NADPH, and 10 mM acetaldehyde was incubated with 4.0 μM of the appropriate epimerase, C4-reductase (4.0 μM), and aldehyde dehydrogenase (2.3 units/mL) in 50 mM phosphate/KOH and 50 mM KCl for 18 h. The reactions were terminated by removing the enzyme from the reaction using a 0.5 mL spin filter with a 10 kDa molecular weight cutoff. The resulting flow-through was injected onto a BioRad FPLC system equipped with a 1.0 mL HiTrap Q HP column. The column was washed with water, and the product was eluted using a linear gradient (0–60%) of 500 mM NH_4_HCO_3_, pH 8.0, over 60 column volumes. Fractions of 0.5 mL were collected and lyophilized to dryness under vacuum. The resulting samples were reconstituted in either D_2_O or H_2_O and analyzed by nuclear magnetic resonance (NMR) spectroscopy and mass spectrometry. All ^1^H NMR and two-dimensional ^1^H-^1^H COSY spectra were recorded on a Bruker Avance III 400 MHz NMR spectrometer at 22 °C. ESI-MS experiments were performed using a Thermo Scientific Q Exactive Focus.

### Reactions Conducted in Oxygen-18 Labeled Water

The proposed reaction mechanisms for the C3-dehydratases and epimerases from HS:5 and HS:11 were tested by conducting the reactions in [^18^O]-H_2_O. The reactions were conducted in a final volume of 100 μL at 22 °C in 50% [^18^O]-H_2_O at pH 7.5 for 18 h. In the first experiment, 4.0 mM GDP-d-*glycero*-α-d-*manno*-heptose (**1**) was incubated with 0.2 mM PLP, 8.0 mM l-glutamate, 4.0 μM C4,6-dehydratase from HS:23/36, and 4.0 μM C3-dehydratase from either HS:5 or HS:11 in 50% [^18^O]-H_2_O in 50 mM phosphate buffer containing 50 mM KCl. In the second experiment 4.0 mM GDP-d-*glycero*-α-d-*manno*-heptose (**1**) was incubated with 0.2 mM PLP, 8.0 mM l-glutamate, 8.0 mM NADPH, 4.0 μM C4,6-dehydratase from HS:23/36, 4.0 μM C3-dehydratase from either HS:5 or HS:11, 4.0 μM of the epimerase from either HS:5 or HS:11, and 4.0 μM of the C4-reductase from either HS:5 or HS:11 in 50% [^18^O]-H_2_O in 50 mM phosphate buffer containing 50 mM KCl. The reactions were terminated by removal of the enzyme using a 0.5 mL spin filter with a 10 kDa molecular weight cutoff, and the products were analyzed by mass spectrometry.

### Determination of Kinetic Constants

All assays, except for the C3-dehydratase, were conducted in a total reaction volume of 250 μL in buffer D (pH 7.5) at 25 °C. The kinetic constants for the reaction catalyzed by the PLP-dependent C3-dehydratase from HS:5 were determined using a coupled enzyme assay by monitoring the formation of α-ketoglutarate (α-KG) with glutamate dehydrogenase at 340 nm. The substrate, GDP-6-deoxy-4-keto-α-d-*lyxo*-heptose (**5**), was initially obtained by incubation of 4.0 mM GDP-d-*glycero*-α-d-*manno*-heptose (**1**) with 4.0 μM C4,6-dehydratase from *C. jejuni* serotype HS:23/36 in buffer D (50 mM HEPES/KOH, pH 7.5) for 2 h at 22 °C. The C4,6-dehydratase was removed using a 3 kDa molecular weight cutoff spin filter. For the determination of the kinetic constants, the concentration of GDP-6-deoxy-4-keto-α-d-*lyxo*-heptose (**5**) was varied between 100 μM and 2.0 mM. The assays were conducted using 2.5 μM C3-dehydratase, 0.2 mM PLP, 4.0 mM l-glutamate, 5 U glutamate dehydrogenase, and 300 μM NADPH in 50 mM ammonium bicarbonate buffer (pH 7.5).

Similarly, the kinetic constants for the reactions catalyzed by the C5-epimerase from HS:5 and the C4-reductases from HS:5 and HS:11 were determined using a coupled enzyme assay by monitoring the oxidation of NADPH to NADP^+^ at 340 nm. For the determination of the kinetic constants, the concentration of GDP-3,6-dideoxy-4-keto-α-d-*threo*-heptose (**8**) was varied between 100 μM and 2.0 mM. For the determination of the kinetic constants of the epimerase from serotype HS:5, the assays were carried out with 50 nM epimerase from HS:5, 20 μM C4-reductase from HS:5, and 300 μM NADPH. For the determination of the kinetic constants for the C4-reductases from either serotype HS:5 or HS:11, the assays were conducted using 10 μM C5-epimerase (HS:5) and 100 or 20 nM C4-reductase from HS:5 or HS:11, respectively, in the presence of 300 μM NADPH. The apparent values of *k*_cat_ and *k*_cat_/*K*_m_ were determined by fitting the initial velocity data to eq 1 using SigmaPlot 11.0, where ν is the initial velocity of the reaction, *E*_t_ is the enzyme concentration, *S* is the substrate concentration, *k*_cat_ is the turnover number, and *K*_m_ is the Michaelis constant.
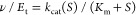
1

### Sequence Similarity Network Analysis of the C3-Dehydratases, Epimerases, and C4-Reductases from *C. jejuni* Serotypes HS:5 and HS:11

The FASTA protein sequences for the C3-dehydratases, epimerases, and C4-reductases from *C. jejuni* serotypes HS:5 and HS:11 were used as the initial BLAST (Basic Local Alignment Search Tool) queries in the EFI-EST database (Enzyme Function Initiative-Enzyme Similarity Tool, https://efi.igb.illinois.edu/efi-est/).^20^ The sequence similarity networks (SSNs) were generated by submitting the FASTA sequences to the EFI-EST webtool. All network layouts were created and visualized using Cytoscape 3.9.1.^21^

## Results and Discussion

### Proposed Biosynthetic Pathway for GDP-3,6-dideoxy-heptoses

The first step in the proposed biosynthesis of GDP-3,6-dideoxy-α-d-*ribo*-heptose (**10**) is the NAD^+^-dependent oxidation/dehydration/reduction of GDP-d-*glycero*-α-d-*manno*-heptose (**1**) by the C4,6-dehydratase.^7−12^ The second step includes removal of the hydroxyl group at C3 from GDP-6-deoxy-4-keto-α-d-*manno*-heptose (**5**) by a PLP-dependent C3-dehydratase and formation of GDP-3,6-dideoxy-4-keto-α-d-*threo*-heptose (**8**). The proposed formation of GDP-3,6-dideoxy-4-keto-α-d-*erythro*-heptose (**9**) occurs via the epimerization of **8** by a C2-epimerase. In the last step, GDP-3,6-dideoxy-α-d-*ribo*-heptose (**10**) is synthesized via the NADPH-dependent reduction of **9** by the C4-reductase. The proposed biosynthetic pathway is summarized in Figure 2.

### Bioinformatic Analysis of the C3-Dehydratase

**Figure 4:**
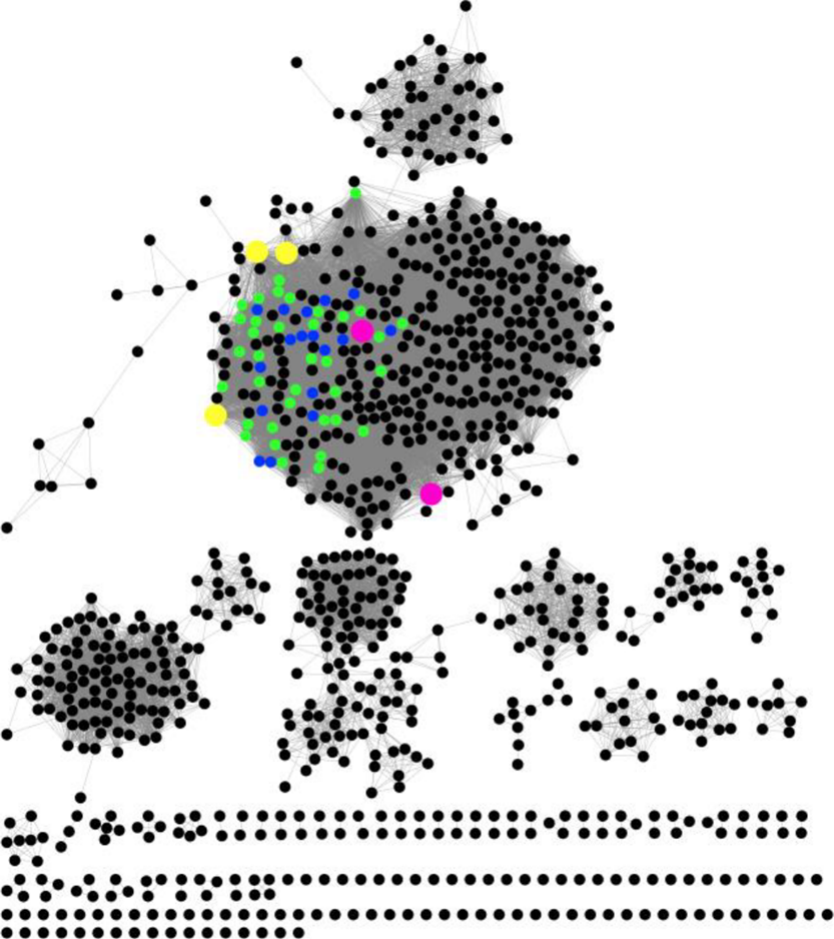
SSN for the C3-dehydratases from *C. jejuni*. The closest 1000 sequences to the C3-dehydratase from *C. jejuni* serotype HS:5 at a sequence identity cutoff of 65%. The sequences for the 3-dehydratases from *E. coli* O55:H7 and *Y. pseudotuberculosis* IVA are shown in pink. The green and blue circles represent the apparent C3-dehydratases from various *Campylobacter* species and *C. jejuni*, respectively. The yellow circles represent the C3-dehydratases from serotypes HS:5, HS:11, and HS:45.

The SSN of the 1000 closest homologues to the proposed C3-dehydratase from *C. jejuni* serotype HS:5 is presented in Figure 4 at a sequence identity cutoff of 65%. The sequences from all *Campylobacter* species are denoted in green, whereas the sequences exclusively identified from *C. jejuni* are colored blue. In this SSN, there are two previously characterized PLP-dependent C3-dehydratases from *E. coli* O55:H7 and *Yersinia pseudotuberculosis* IVA.^22,23^ These two enzymes have been shown to catalyze the loss of the hydroxyl group at C3 from GDP-6-deoxy-4-keto-α-d-mannose to form GDP-3,6-dideoxy-4-keto-α-d-mannose.^22,23^ The three previously uncharacterized C3-dehydratases from *C. jejuni* are found in the HS:5, HS:11, and HS:45 serotypes of *C. jejuni*. The C3-dehydratases from these three serotypes are >94% identical to one another and 62–67% identical to the two previously characterized PLP-dependent C3-dehydratases from *E. coli* O55:H7 and *Y. pseudotuberculosis* IVA.

### Isolation and Functional Characterization of C3-Dehydratase

We isolated the two putative PLP-dependent C3-dehydratases from the HS:5 and HS:11 serotypes of *C. jejuni*. The C3-dehydratases were produced in *E. coli* BL21(DE3) cells with a 21-residue His_6_-containing affinity tag appended to the N-terminus. The enzymes were purified to homogeneity using metal affinity chromatography. After purification, there were no bound cofactors in the as-isolated proteins.

### Reaction Catalyzed by the C3-Dehydratase

**Figure 5:**
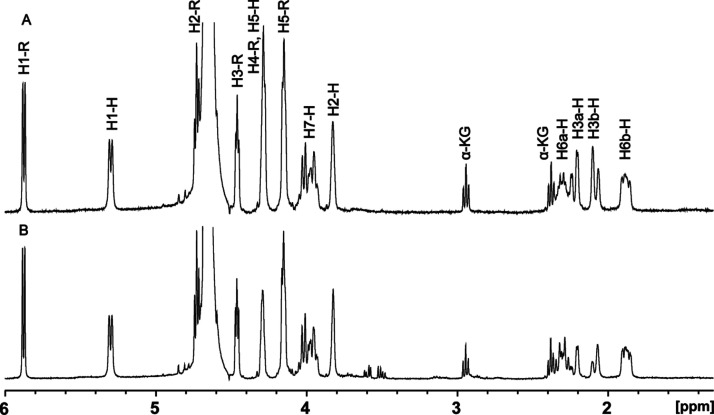
^1^H NMR spectra of GDP-3,6-dideoxy-4-keto-α-d-*threo*-heptose (**8**) produced with the C3-dehydratase from serotype HS:5. (A) Reaction conducted in H_2_O. (B) Reaction conducted in D_2_O. Resonances for the hydrogens labeled with an “R” correspond to the ribose moiety of GDP, while those labeled with an “H” correspond to those of the heptose moiety. The multiplet *a* ∼3.6 ppm is likely due to contamination of glycerol. α-KG is the other reaction product formed from l-glutamate. Additional details are provided in the text.

**Figure 6:**
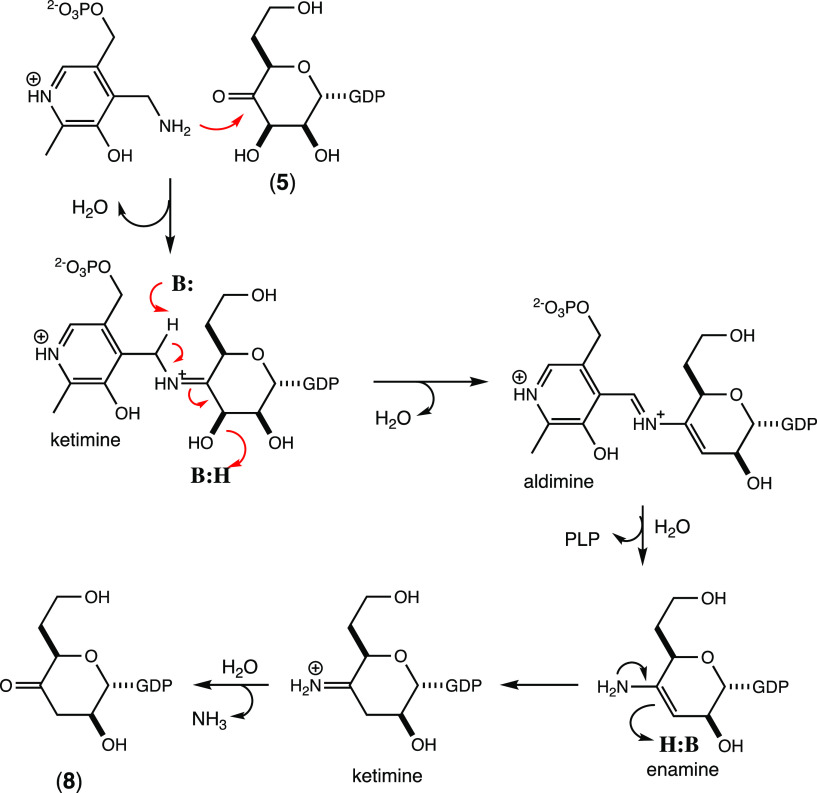
Proposed reaction mechanism for the PLP-dependent C3-dehydratase. The PLP is recycled back to PMP in the first half reaction with l-glutamate.

We investigated the reaction catalyzed by the C3-dehydratase using GDP-6-deoxy-4-keto-α-d-*lyxo*-heptose (**5**). When this substrate was incubated with the C3-dehydratase from either serotype HS:5 or HS:11 in the presence of PLP and l-glutamate, a new compound was formed whose ^1^H NMR spectra using the enzyme from serotype HS:5 are provided in Figures 5a, S2, and S3. In the new product, the two hydrogens attached to C3 are clearly apparent at 2.23 and 2.09 ppm (Figure 5a). When the reaction is conducted in D_2_O, the resonance for the hydrogen attached to C5 disappears because it has been exchanged for deuterium from the solvent due to the catalytic activity of the C4,6-dehydratase used in the preparation of compound **5** from compound **1** (Figure 5b). The two hydrogens attached to C3 lost ∼50% of their original intensity when the reaction was conducted in D_2_O. This observation suggests that the hydrogen from the solvent that replaces the hydroxyl group at C3 has been added nonstereospecifically (see the proposed reaction mechanism in Figure 6). An identical product was formed using the C3-dehydratase from serotype HS:11, and the NMR spectra are shown in Figures S4 and S5. The ^1^H NMR chemical shifts for the isolated products are summarized in Table 1.

**Table 1:** ^1^H NMR Chemical Shifts for GDP-heptoses Prepared for This Investigation

enzyme serotype	heptose structure	chemical shifts, ppm						
		H1	H2	H3	H4	H5	H6	H7
HS:5	GDP-3,6-dideoxy-4-keto-α-d-*threo*-heptose (**8**)	5.31	3.83	2.23, 2.09	na	4.29	2.31, 1.89	3.99
HS:11	GDP-3,6-dideoxy-4-keto-α-d-*threo*-heptose (**8**)	5.31	3.83	2.23, 2.09	na	4.29	2.31, 1.89	3.99
HS:5	GDP-3,6-dideoxy-β-l-*ribo*-heptose (**12**)	4.86	3.45	2.30, 1.56	3.38	3.44	2.01, 1.48	3.69
HS:11	GDP-3,6-dideoxy-β-l-*xylo*-heptose (**13**)	4.87	3.67	2.16, 1.67	3.78	3.67	1.80, 1.67	3.67
HS:2	GDP-3,6-dideoxy-β-l-*ribo*-heptose (**12**)	4.86	3.45	2.30, 1.67	3.38	3.44	2.01, 1.48	3.69
HS:15	GDP-3,6-dideoxy-β-l-*xylo*-heptose (**13**)	4.87	3.67	2.16, 1.67	3.78	3.67	1.80, 1.67	3.67
HS:53	GDP-3,6-dideoxy-α-d-*arabino*-heptose (**14**)	5.25	3.93	1.95, 1.82	3.66	3.81	2.00, 1.84	3.66
HS:3	GDP-3,6-dideoxy-α-d-*lyxo*-heptose (**15**)	5.38	3.77	2.05, 1.86	3.65	4.12	1.80, 1.66	3.65

**Figure 7:**
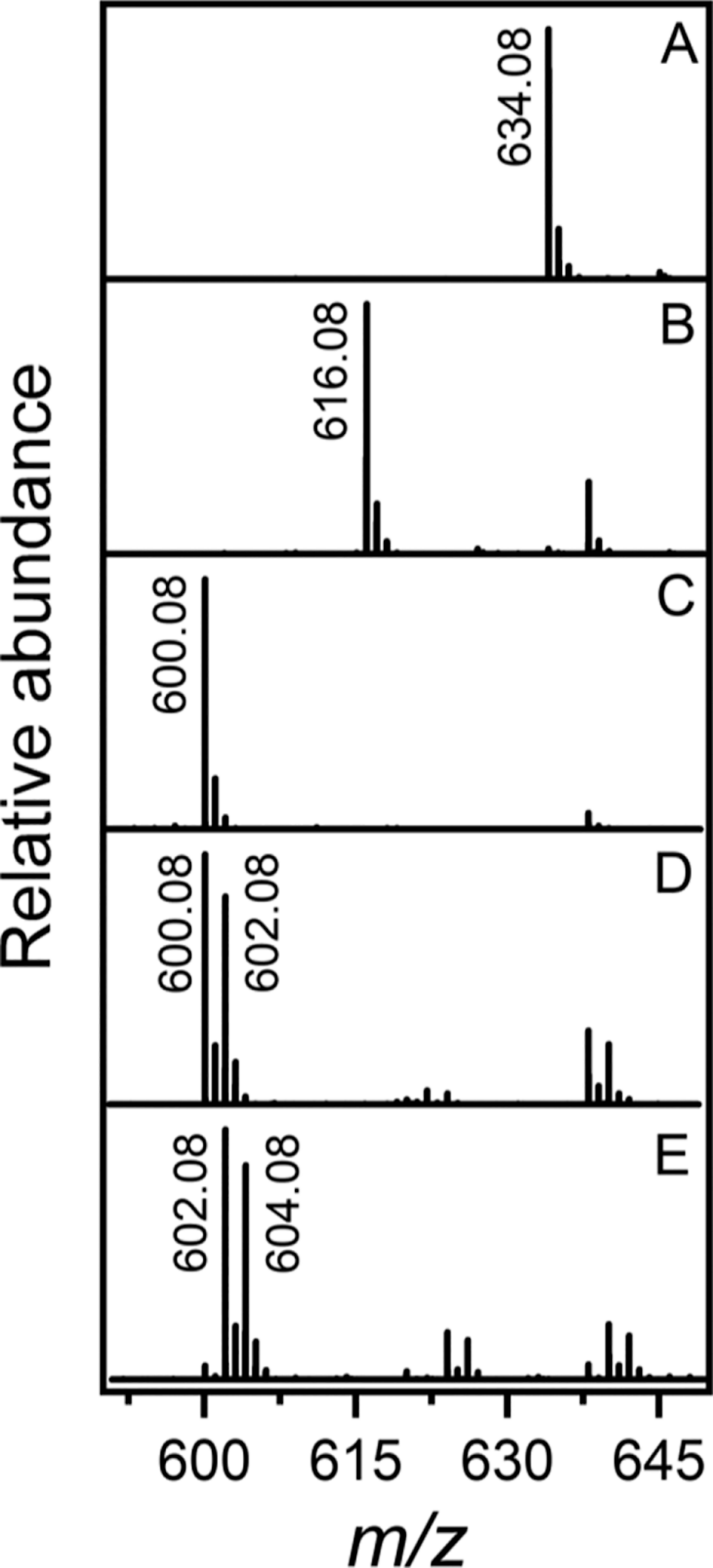
(A) Mass spectrometry analysis of the reaction catalyzed by the C3-dehydratase. GDP-d-*glycero*-α-d-*manno*-heptose (**1**) prior to the addition of enzyme. (B) Reaction product, GDP-6-deoxy-4-keto-α-d-*lyxo*-heptose (**5**), after the addition of the C4,6-dehydratase to compound **1**. (C) Reaction product, GDP-3,6-dideoxy-4-keto-d-*threo*-heptose (**8**), after the addition of the C3-dehydratase from serotype HS:5 to compound **5**. Reaction products **5** and **8** appear at an *m*/*z* of 616.08 and 600.08, respectively. (D) Reaction product, GDP-3,6-dideoxy-4-keto-α-d-*threo*-heptose (**8**), after the addition of the C3-dehydratase from serotype HS:5 to compound **5** in 50% [^18^O]-H_2_O. (E) Reaction product, GDP-3,6-dideoxy-β-l-*ribo*-heptose (**12**), produced from compound **1** by the enzymatic activities of the C4,6-dehydratase, C3-dehydratase, and C4-reductase in 50% [^18^O]-H_2_O.

The reaction products were confirmed using mass spectrometry. The ESI-MS (negative ion mode) of GDP-d-*glycero*-α-d-*manno*-heptose (**1**) before the addition of the C4,6-dehydratase is shown in Figure 7a with an *m/z* of 634.08 for the M-H anion. The ESI-MS of GDP-6-deoxy-4-keto-α-d-*lyxo*-heptose (**5**) at an *m*/*z* of 616.08 for the M-H anion was obtained after incubation of **1** with the C4,6-dehydratase (Figure 7b). When the C3-dehydratase reaction was conducted in H_2_O, the ESI-MS of the product exhibits an *m/z* of 600.08 for the M-H anion, consistent with the loss of one oxygen atom and the formation of GDP-3,6-dideoxy-4-keto-α-d-*threo*-heptose (**8**), as shown in Figure 7c. When the C3-dehydratase reaction was conducted in 50% [^18^O]-H_2_O, product **8** exhibited nearly identical peaks at an *m*/*z* of 600.08 and 602.08, consistent with the incorporation of one oxygen from the solvent at C4 (Figure 7d). These experiments clearly demonstrate that the C3-dehydratases from serotypes HS:5 and HS:11 catalyze the PLP-dependent conversion of GDP-6-deoxy-4-keto-α-d-*manno*-heptose (**5**) to GDP-3,6-dideoxy-4-keto-α-d-*threo*-heptose (**8**).

### Reaction Mechanism for the PLP-Dependent C3-Dehydratase

The reaction mechanism for the PLP-dependent C3-dehydratase from *E. coli* O55:H7 and *Y. pseudotuberculosis* IVA has previously been addressed by the Holden and Liu laboratories.^22,23^ A similar transformation can be postulated for the C3-dehydratase from *C. jejuni*. In the first half of the reaction mechanism, the enzyme uses l-glutamate to convert PLP to pyridoxamine 5′-phosphate (PMP) and α-KG (not shown). In the second half of the reaction mechanism, the primary amino group of PMP forms a ketimine intermediate with the C4 carbonyl group of compound **5** with the loss of water. In the next step, a general base in the active site abstracts a proton from the methylene group of PMP with the subsequent expulsion of the hydroxyl group at C3 to generate an aldimine intermediate. Hydrolysis of this intermediate releases PLP with formation of an enamine intermediate. Subsequent hydrolysis of the enamine intermediate results in the ultimate formation of GDP-3,6-dideoxy-4-keto-α-d-*threo*-heptose (**8**) and ammonia. This reaction mechanism clearly explains the incorporation of an ^18^O from labeled water at C4, and it also suggests that hydrolysis of the enamine intermediate occurs after release into solution since the incorporation of deuterium from the solvent water at C3 appears to be nonstereospecific.

### Bioinformatic Analysis of the Uncharacterized Epimerase from Serotypes HS:5, HS:11, and HS:45

**Figure 8:**
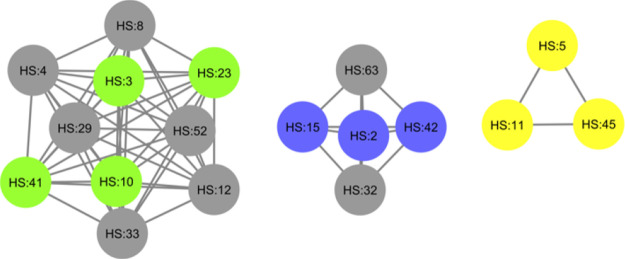
SSN for 18 epimerases identified from 33 serotyped strains of *C. jejuni* at a sequence identity of 89%. The specific serotype is labeled in each circle. The nodes in green and blue colors represent the C3- and C3/C5-epimerases, respectively, that were previously tested and functionally annotated for catalytic activity,^13^ whereas the yellow color designates the epimerases from serotypes HS:5, HS:11, and HS:45 of unknown function.

We identified 18 GDP-heptose epimerases within the gene clusters utilized for the biosynthesis of CPSs in 33 serotyped strains of *C. jejuni*. At a sequence identity of 89%, these 18 epimerases cluster together within the SSN into three well-defined groups (Figure 8). The largest group has been functionally characterized as being able to epimerize C3 using GDP-6-deoxy-4-keto-α-d-*lyxo*-heptose (**5**) as the substrate, and the second largest group has been shown to epimerize both C3 and C5 from the same substrate.^13^ The three epimerases from the smallest cluster are found juxtaposed next to a C3-dehydratase in the associated gene clusters and thus likely involved in the formation of the 3,6-dideoxy-heptoses in *C. jejuni* (see the gene cluster for the HS:5 serotype in Figure 3). A multiple sequence alignment of the 18 epimerases from the serotyped strains of *C. jejuni* is shown in Figure S6. All of these enzymes contain a fully conserved dyad of histidine and tyrosine required for utilization as general acid/base catalysts for the abstraction and addition of hydrogen ions from the substrate during the isomerization process.^9−11,13^

### Determination of Epimerase-Catalyzed Reaction

In the proposed biosynthetic reaction mechanism for the formation of GDP-3,6-dideoxy-α-d-*ribo*-heptose (**10**), the epimerase is used to isomerize the stereochemistry at C2 prior to reduction of the carbonyl group at C4 (Figure 2). Isomerization of C2 could be accomplished via the oxidation/reduction of C2 via an NAD^+^/NADH cycle or through the loss of water from C3/C2 followed by rehydration on the opposite side of the newly formed double bond.^24^ The epimerase identified within the gene cluster from serotype HS:5 is highly unlikely to be capable of an NAD^+^-dependent oxidation/reduction but is more likely to be able to initiate the loss of water at C3/C2 via proton abstraction at C3, coupled with the loss of the hydroxyl group at C2, followed by rehydration of the double bond. However, this type of isomerization at C2 has not been previously described for the family of isomerases identified here.

In order to more fully characterize the molecular details of the epimerase-catalyzed reaction, GDP-d-*glycero*-α-d-*manno*-heptose (**1**) was incubated with the C4,6-dehydratase, C3-dehydratase, epimerase, and the C4-reductase from the HS:5 serotype in the presence of PLP, l-glutamate, and NADPH in 50% [^18^O]-H_2_O. The unknown GDP-3,6-dideoxy-heptose product was isolated, and the ESI mass spectrum was obtained, showing peaks at an *m/z* of 602.08 and 604.08 with an intensity ratio of ∼1:1 (Figure 7e). If epimerization at C2 occurred via a dehydration/rehydration mechanism, then we should have observed the incorporation of ^18^O at both C2 and C4, resulting in peaks at *m/z* of 602.08, 604.08, and 606.08 with an intensity ratio of ∼1:2:1. However, the observed peaks found at an *m*/*z* of 602 and 604 are consistent only with the exchange of ^18^O at C4 due to the action of the C3-dehydratase.

It is also possible for an epimerization to occur via an E1 elimination mechanism where GDP departs from compound **8** to form an oxocarbenium intermediate. Abstraction of the C2 proton by an active site base would then form a glycal intermediate, which is subsequently reprotonated on the opposite side with formation of the epimeric product. This transformation is similar to that proposed by Tanner for the reaction catalyzed by UDP-*N*-acetylglucosamine 2-epimerase.^25,26^ However, this mechanism requires the exchange of the hydrogen at C2 with solvent deuterium when the reaction is conducted in D_2_O, but no exchange at C2 is apparent under these conditions (vide infra). Therefore, it is highly unlikely that any of the epimerases from serotypes HS:5, HS:11, or HS:45 are functionally able to epimerize C2.

### NMR Analysis of the Epimerase Reaction Product

**Figure 9:**
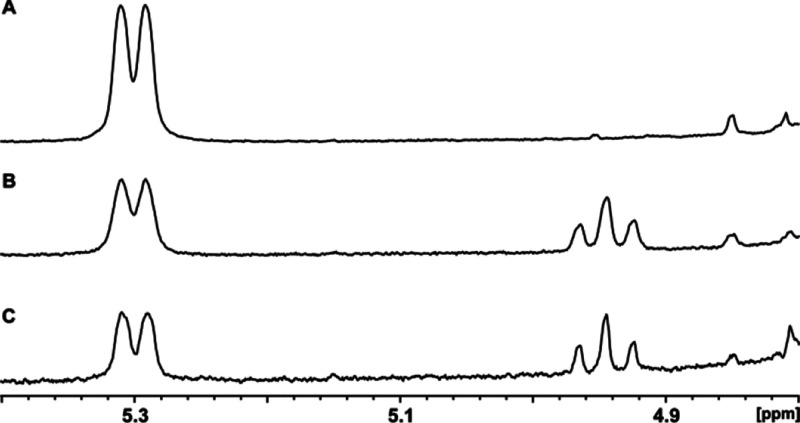
Portion of the ^1^H NMR spectra showing the anomeric hydrogen at C1 of products formed from GDP-3,6-dideoxy-4-keto-α-d-*threo*-heptose (**8**) after the addition of an epimerase from either serotype HS:5 or HS:2. (A) GDP-3,6-dideoxy-4-keto-α-d-*threo*-heptose (**8**), (B) GDP-3,6-dideoxy-4-keto-α-d-*threo*-heptose (**8**) after the addition of epimerase from serotype HS:5, and (C) GDP-3,6-dideoxy-4-keto-α-d-*threo*-heptose (**8**) after the addition of an epimerase from serotype HS:2. The new triplet at 4.94 ppm is from the anomeric hydrogen at C1 of the product GDP-3,6-dideoxy-4-keto-β-l-*erythro*-heptose (**11**).

**Figure 10:**
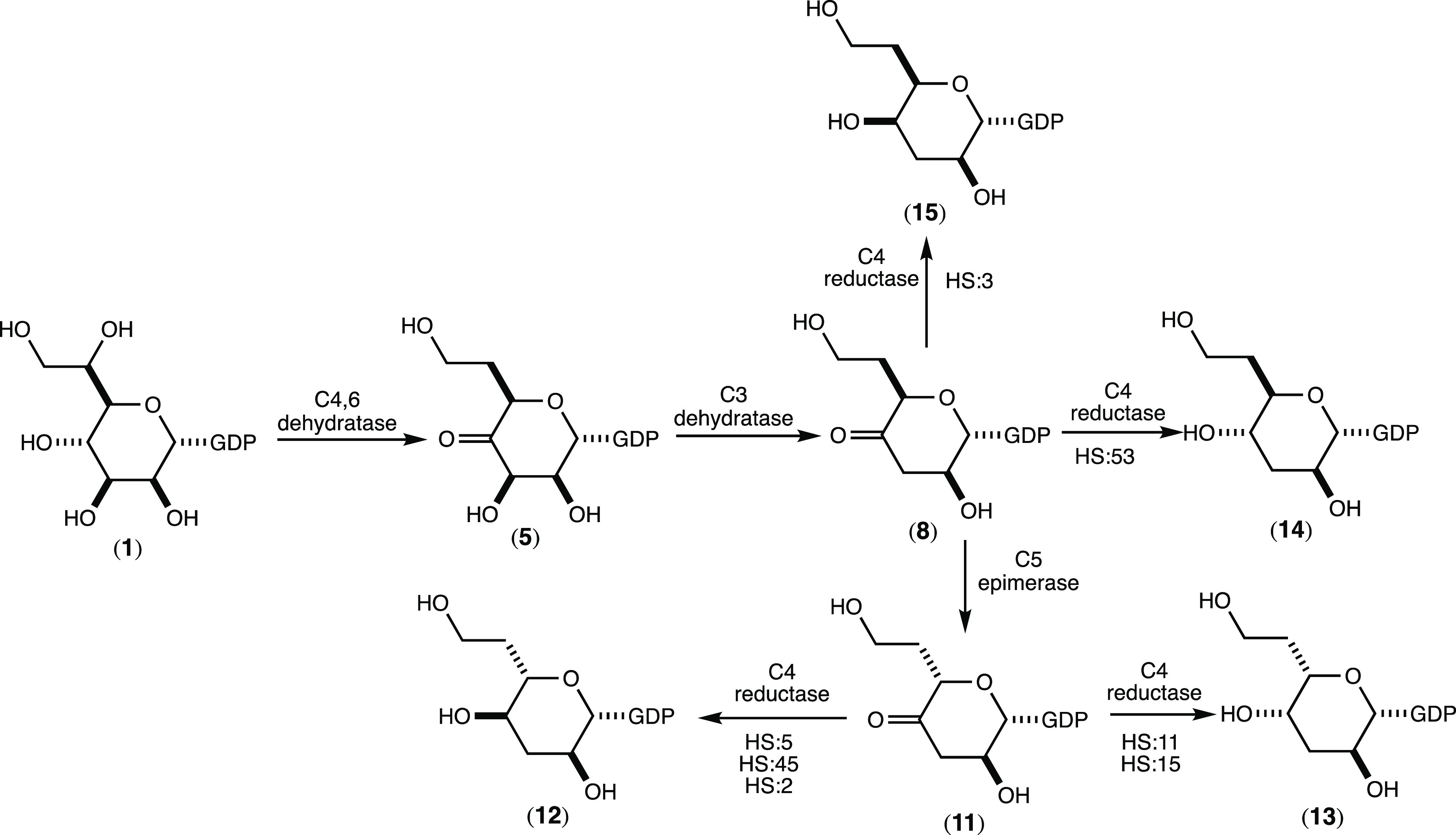
New biosynthetic pathways for the formation of GDP-3,6-dideoxy-β-l-*ribo*-heptose (**12**) and GDP-3,6-dideoxy-β-l-*xylo*-heptose (**13**).

**Figure 11:**
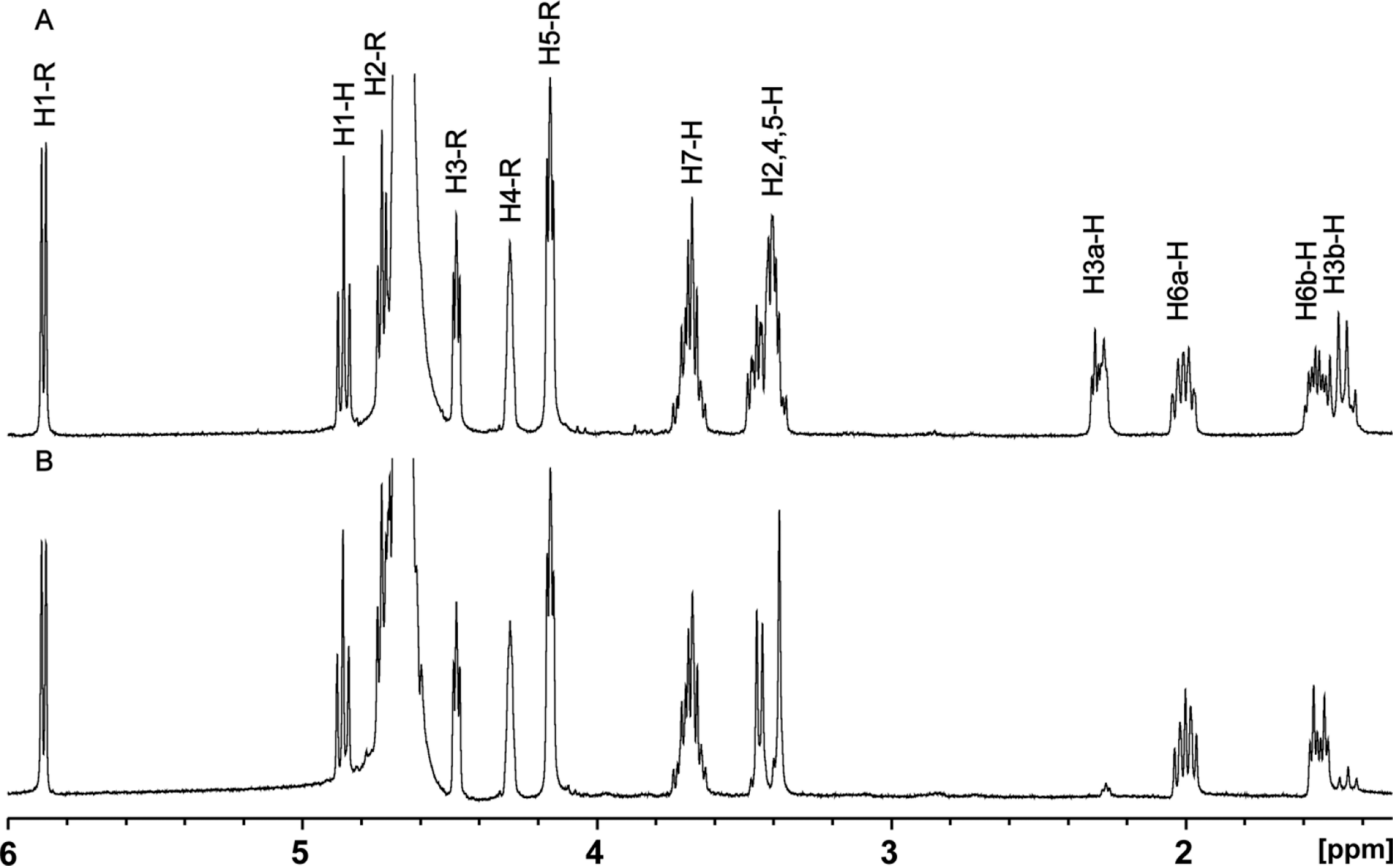
^1^H NMR spectra of GDP-3,6-dideoxy-β-l-*ribo*-heptose (**12**) using the C4-reductase from serotype HS:5. (A) Reaction conducted in H_2_O. (B) Reaction conducted in D_2_O. The loss of the resonances for C3 when the reaction was conducted in D_2_O likely reflects the combined activities of the C3-dehydratase and the C5-epimerase used in the preparation of compound **12**. Resonances for the hydrogens labeled with an “R” correspond to the ribose moiety of GDP, while those labeled with an “H” correspond to those of the heptose moiety. Additional details are provided in the text.

When GDP-3,6-dideoxy-4-keto-α-d-*threo*-heptose (**8**) is incubated with the epimerase from either serotype HS:5 or HS:11, a new triplet appears at 4.94 ppm for the hydrogen at C1 in the ^1^H NMR spectrum (Figure 9). The new triplet indicates that the stereochemistry at C5 has been epimerized since the previous ^18^O experiment demonstrates that the epimerase is functionally unable to change the stereochemistry at C2. An equilibrium constant for the formation of compound **11** (Figure 10), [**11**]/[**8**], of 0.63 ± 0.1 was calculated based on the relative intensities of the hydrogen at C1 for the substrate (**8**) and the newly formed epimerized product (**11**). When compound **8** was incubated with the functionally characterized C3/C5-epimerase from serotype HS:2,^13,16^ the NMR spectrum of the product (**11**) was virtually the same as when the epimerase from HS:5 was used, demonstrating that they form the same reaction product. The proposed transformation is shown in Figure 10, and thus, the epimerase from serotypes HS:5, HS:11; and HS:45 can be designated as a C5-epimerase that catalyzes the epimerization of the stereochemistry at C5 within GDP-3,6-dideoxy-4-keto-α-d-*threo*-heptose (**8**) to GDP-3,6-dideoxy-4-keto-β-l-*erythro*-heptose (**11**).

### Bioinformatic Analysis of C4-Reductases from HS:5, HS:11, and HS:45

We previously identified 25 C4-reductases from 33 different serotyped strains of *C. jejuni*.^14,15^ The SSN analysis for the 25 C4-reductases presented in Figure S7 indicates that at a sequence identity of 89%, nine distinct groups are apparent.^14^ The C4-reductases from HS:5 and HS:45 belong to the same group and likely catalyze the formation of identical products, whereas the C4-reductase from HS:11 forms a separate group. The sequence identity between the C4-reductases from HS:5 and HS:45 is 96% but only 41–44% identical to that from HS:11.

### Reactions Catalyzed by the C4-Reductases from Serotyped Strains HS:5 and HS:11 of *C. jejuni*

We investigated the reactions catalyzed by the C4-reductases from HS:5 and HS:11 using GDP-3,6-dideoxy-4-keto-α-d-*threo*-heptose (**8**) as the starting substrate with the appropriate epimerase. When compound **8** is incubated with the epimerase and C4-reductase from serotype HS:5 in the presence of NADPH, a new compound is formed whose ^1^H NMR spectra are provided in Figures 11a and S8. If the reactions are conducted in D_2_O, the resonances for the hydrogens attached to C3 and C5 disappear in the ^1^H NMR spectrum because they have been exchanged for deuterium from the solvent due to the catalytic activities of the C4,6-dehydratase and the C5-epimerase used in the preparation of compound **8** in D_2_O (Figures 11b and S9). The assignment of resonances in the NMR spectra is based on the 2D COSY NMR spectrum and the loss of signals for the hydrogens at C3 and C5 when the product is made in D_2_O.

When GDP-3,6-dideoxy-4-keto-α-d-*threo*-heptose (**8**) is incubated with the C5-epimerase and C4-reductase from serotype HS:11 in the presence of NADPH, a new compound is formed whose ^1^H NMR spectrum is distinctly different from that observed using the C4-reductase from HS:5. The ^1^H NMR spectrum for the product formed in H_2_O is presented in Figure S10 and that in D_2_O is shown in Figure S11. Similar experiments were conducted using the C3/C5-epimerase from HS:2 and the C4-reductase from either HS:2 or HS:15. When the C4-reductase from HS:2 is utilized, the ^1^H NMR spectrum of the final product is identical to that of the C4-reductase product from HS:5 (compare Figure 11a with Figure S12). Conversely, the ^1^H NMR spectrum of the product formed from the addition of the C4-reductase from HS:15 is identical to that of the C4-reductase product from HS:11 (compare Figure S10 with Figure S13). These results show conclusively that the product formed from the addition of the C5-epimerase and the C4-reductase from HS:5 is GDP-3,6-dideoxy-β-l-*ribo*-heptose (**12**) and that the product formed from the addition of the C5-epimerase and C4-reductase from HS:11 is GDP-3,6-dideoxy-β-l-*xylo*-heptose (**13**). These assignments are based on the known C4-reductase products from HS:2 and HS:15, and it is now apparent that these two C4-reductases are functionally able to catalyze the reduction of 3-deoxy substrates.^14,15^

### Preparation of Previously Uncharacterized GDP-3,6-dideoxy-heptoses

From the experiments denoted above, it is apparent that the C4-reductases from serotypes HS:2 and HS:15 can catalyze the reduction of the C4-keto group in GDP-3,6-dideoxy-4-keto-β-l-*erythro*-heptose (**11**) to generate either **12** or **13**. We therefore prepared GDP-3,6-dideoxy-4-keto-α-d-*threo*-heptose (**8**) by the action of the C4,6-dehydratase and the C3-dehydratase and subsequently added the C4-reductases from either HS:3 or HS:53. In each case, a new product was formed, and the NMR spectrum for each product is distinct from that obtained for either compound **12** or **13** (Figures S14 and S15). Based on the known product outcomes for the C4-reductase from HS:53 and HS:3, the two new products are GDP-3,6-dideoxy-α-d-*arabino*-heptose (**14**) and GDP-3,6-dideoxy-α-d-*lyxo*-heptose (**15**), respectively (Figure 10).

### Steady-State Kinetic Constants

The kinetic constants for the C3-dehydratase from serotype HS:5 were determined by monitoring the oxidation of NADPH to NADP^+^ at 340 nm. The C3-dehydratase forms α-KG as a reaction product during the reaction with the substrate GDP-6-deoxy-4-keto-α-d-*lyxo*-heptose (**5**). The formation of α-KG was quantified by glutamate dehydrogenase. The kinetic constants are provided in Table 2. The catalytic activities of the C5-epimerase from HS:5 were determined using a coupled assay with an excess of the C4-reductase from serotype HS:5 by monitoring the oxidation of NADPH as a function of time. The substrate used for the C5-epimerase was GDP-3,6-dideoxy-4-keto-α-d-*threo*-heptose (**8**). The kinetic constants are provided in Table 2. Similarly, the kinetic constants for the C4-reductases from serotypes HS:5 and HS:11 were determined by the NADPH-dependent reduction of the substrate GDP-3,6-dideoxy-4-keto-α-d-*threo*-heptose (**8**) in the presence of an excess of the C5-epimerase. The steady-state concentration of the substrate, GDP-3,6-dideoxy-4-keto-α-d-*threo*-heptose (**8**), was calculated from the equilibrium constant for the reaction of the C5-epimerase with GDP-3,6-dideoxy-4-keto-α-d-*threo*-heptose (**8**). The kinetic constants are provided in Table 2.

**Table 2:** Steady-State Kinetic Parameters for C3-Dehydratase, C5-Epimerase, and C4-Reductases^a^

enzyme	*k*_cat_ (s^–1^)	*K*_m_ (μM)	*k*_cat_/*K*_m_ (M^–1^ s^–1^)
C3-dehydratase (HS:5)	0.17 ± 0.01	840 ± 60	203 ± 8
C5-epimerase (HS:5)	3.1 ± 0.3	480 ± 80	6400 ± 600
C4-reductase (HS:5)	1.1 ± 0.2	470 ± 90	2400 ± 200
C4-reductase (HS:11)	5.7 ± 0.6	870 ± 160	6500 ± 500

a pH = 7.5, 25 °C.

## Conclusions

The biosynthetic pathway for the assembly of 3,6-dideoxy-heptoses from the human pathogen *C. jejuni* was determined. We identified four genes in the operon for CPS biosynthesis in the HS:5 serotype of *C. jejuni* that were used to convert GDP-d-*glycero*-α-d-*manno*-heptose (**1**) to GDP-3,6-dideoxy-β-l-*ribo*-heptose (**12**). In the first step, **1** is converted to GDP-6-deoxy-4-keto-α-d-*lyxo*-heptose (**5**). This product is then dehydrated by a PLP-dependent C3-dehydratase to form GDP-3,6-dideoxy-4-keto-α-d-*threo*-heptose (**8**) before being epimerized at C5 to generate GDP-3,6-dideoxy-4-keto-β-l-*erythro*-heptose (**11**). In the final step, a C4-reductase uses NADPH to convert product **11** to **12**. These results are at variance with the identification of 3,6-dideoxy-d-*ribo*-heptose in the CPS of *C. jejuni* from serotype HS:5. The same set of genes is found in the HS:45 serotype of *C. jejuni*, and thus, it is highly likely that 3,6-dideoxy-β-l-*ribo*-heptose will be found in the CPS of this serotype. In the HS:11 serotype, we have demonstrated that the final product is GDP-3,6-dideoxy-β-l-*xylo*-heptose (**13**).

## Supporting Information Available

Amino acid sequences of the purified proteins; multiple sequence alignment for the epimerases; sequence similarity network for 25 C4-reductases; and NMR spectra of carbohydrate substrates and products (PDF)

## Accession Codes

C3-dehydratases from serotypes HS:5 (A0A0U3ALB0) and HS:11 (A0A0U2RG51); C5-epimerase from HS:5 (A0A0Q3UFL0) and HS:11 (A0A0U2QGV6); and the C4-reductases from HS:5 (A0A0U3C2F2) and HS:11 (A0A0U3ANW2).
