# Supplementary material for: Biosynthesis of 3,6-Dideoxy-heptoses for the Capsular Polysaccharides of Campylobacter jejuni
Source: Biochemistry. 2023 Mar 21;62(7):1287–97. doi: 10.1021/acs.biochem.3c00012 (PMC10440746; doi:10.1021/acs.biochem.3c00012)
Supplement: Supplementary file 1 — bi3c00012_si_001.pdf [file bi3c00012_si_001.pdf]

Supporting Information

Biosynthesis of 3,6-dideoxy-heptoses for the Capsular Polysaccharides of *Campylobacter jejuni*

Manas K. Ghosh, Dao Feng Xiang, and Frank M. Raushel*

Department of Chemistry, Texas A&M University,
College Station, Texas 77845 US

To whom correspondence may be sent: raushel@tamu.edu

(A)
MGSSHHHHHHSSGLVPRGSHMMKYTLASSTWDEKELQAIQDVIKSDMFTMGKKVAEFEKDFAKFTGSRY
AVMTSSGSAANLIATAALFYTKNPKLTRGDEVIVPAVSWSTTYYPFYQYGLKLKFVDIDLETLNYNLDALSS
AISDRTKMIMVVNLLGNPNDFDAINNLIKGKDIILLEDNCESMGAEYKGKQTGTFGIMGTFSTFFSHHMAT
MEGGFVITDDEELYHILLCLRAHGWTRNLPKENLVANKSDDWFSESFRFVLPGYNVRPVEMSGAIGVEQLK
KLPMFLKHRRENAKLFCEYFQNHPEFIMQKEIGSSSWFGFSLVIRPNSKLQRKDIIKKLEENDIEYRPIATGD
FTQNEVIKYFDYEIHQDLKNAKYIHEKGFFVGNHQFSISEQISLFKKVLDNL

(B)
MGSSHHHHHHSSGLVPRGSHMMKKYTLASSTWDEKELQAIQDVIKSDMFTMGKKVAEFEKDFAKFVGSK
YAVMTSSGSTANLIATAALFYTKNPKLKRGDEVIVPAVSWSTTYYPLYQYGLKLKFVDIDLETLNYDLEALSS
AISDKTKMIMVVNLLGNPNDFDAINDLIKGKDIILLEDNCESMGAEYKGNQAGTFGIMGTFSTFFSHHMAT
MEGGFVVTDDEELYHILLCLRAHGWTRNLPKENLVANKSDDWFSESFRFVLPGYNVRPVEMSGAIGIEQLK
KLPMFLKHRRENAKLFCEYFQNHPEFIIQKEIGSSSWFGFSLVIRPNSKLQRKDIIKKLEENGIEYRPIATGDF
TQNEVIKYFDYEIHNELKNAKYIHERGFFVGNHQFSISEQINLFKKVLDNL

(C)
MGSSHHHHHHSSGLVPRGSHMMAIEFDIQESKILKGVYIITPNKFRDLRGEIWSAFSEDCLKHLIPDNLNFV
LDKFTLSKPNVLRGIHGDHKSWKLVTCVYGEVHQVVVDCRKDSPTYLKWEKFIINQNNQKLILIPPYFGNA
QYVSSKSDALYYYKWAYEGEYVDAKEQFTYAWNDSRIAIDWPTNNPILSDRDIDATINDHTKGF

(D)
MGSSHHHHHHSSGLVPRGSHMMAIEFDIQESKILKGVFIITPNSFKDLRGEIWSVFSSKDIESLLPSNLKFVL
DKFTLSKHNVLRGIHGDHKSWKLVTCVYGEVHQVVVDCRKDSPTYLKWERFIINENNRKLILIPPYFGNAQ
YVSSKNDALYYYKWAYEGEYVDAKDQFTYAWNDSRIAIDWSTDTPILSDRDIDATINDHIKG

(E)
MGSSHHHHHHSSGLVPRGSHMMNKDSRIYIAGHKGTAGTALVENLKKRGYENLILKTRQELDLLNQQAVIE
FFKNEQPEYVFLAAVLPCGAANVSQRADFIYENITIQNNIIHQSFKFGVKKLVFFGSGYMYPEKTLNPIKEES
MLTDILEYNATSFGVAKISGTLMCESYNIQYGTNFITLALNNLYGTRANFDFGKSRVLPALLRKFHLAKLLEE
EREDEILKDLKMKSFVEAKKDLTDFGILKDCVEIWGTGKVRREFIHSDDLADAAIYVMENVNFSDLYKKDE
KIKNTHINIGTGIDYSIAEVAQMVKQIVGFKGKLIFNPNRPDSTMDRLMDCSKIHSLGWRHKIELKDGIKMM
YDWYLNKGE

(F)
MGSSHHHHHHSSGLVPRGSHMMNKQSKIYVAGHTGLIGSTILKKLQQDGFCNIICKTHQELDLMNQEAVKY
FFEKEKPDCVFFCAAKVGGMLAQLNQRAEFLYNNLVMQSNVIHYSYLNGVKKLIYLGSICIYPEEVQLPIKES
SLLTGKLQYNNEPYAIAKIAGLKMCEFYSLQYGVDYISIMPVSIYGSNDNFDIQTAHVQAAIFRKIYLAKLLNE
CNYKELIDDLGVKTKEEAFMILKQNNINDKSVKLLGTGNSRREFLHCEDLAEASIYIMKNISFNEVVEREGW
NQGNAHINIGTGEDISIKELAYMIKDILEYRGEVLFENKMENDGTARKVIDTNKIRALGWHYKIELRDGLVR
MYHDYKKGVKQNEKIYTSFINLG

Figure S1. Sequences of the proteins from *Campylobacter jejuni* purified for this investigation. These enzymes include the C3-dehydratases from serotype HS:5 (A) and HS:11 (B); epimerases from serotypes HS:5 (C) and HS:11 (D); and the C4-reductases from serotypes HS:5 (E) and HS:11 (F). The residues added by the purification tags are highlighted in red.

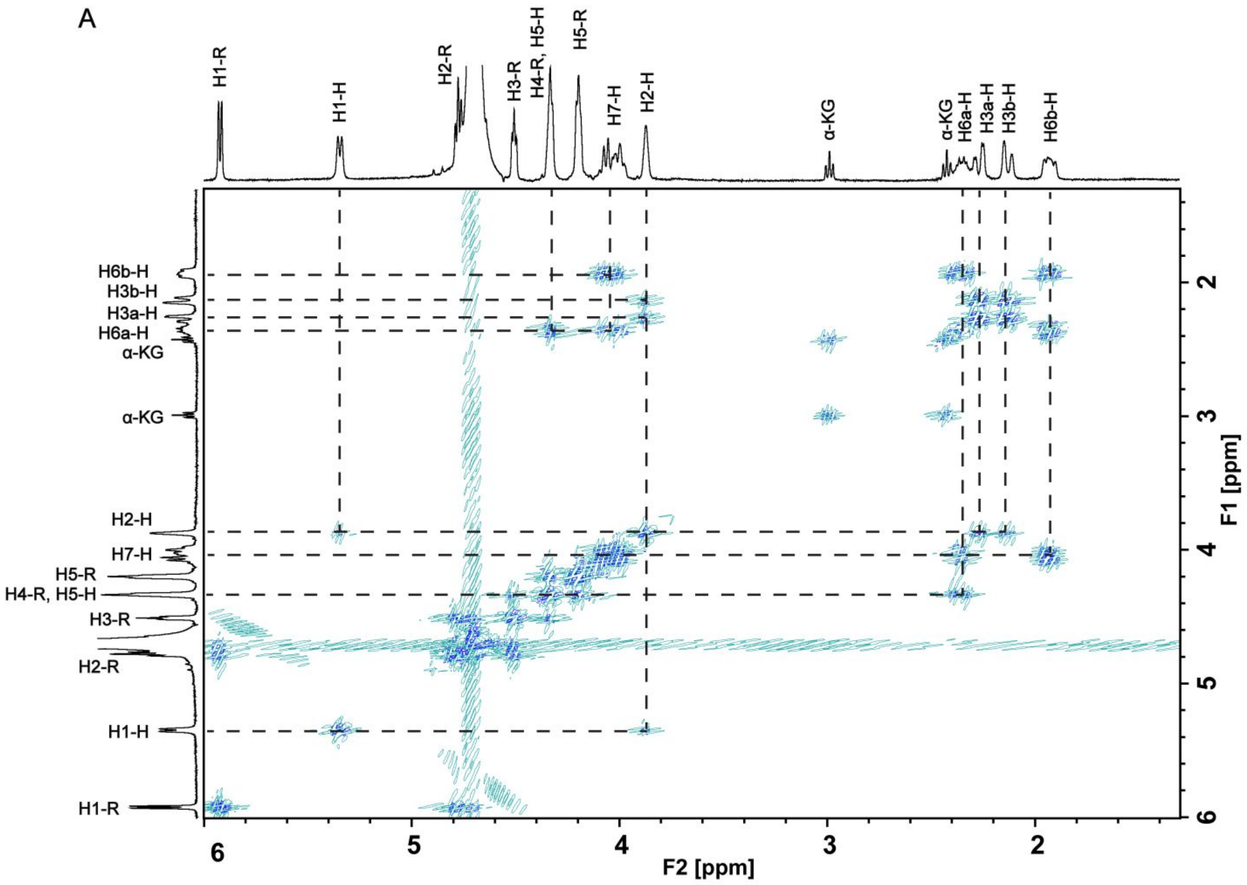

Figure S2: ^{1}H-^{1}H COSY NMR spectrum of GDP-3,6-dideoxy-4-keto-α-D-*threo*-heptose (**8**) prepared in H_2O using the C3-dehydratase from serotype HS:5. Resonances for the hydrogens labeled with an ''R'' correspond to the ribose moiety of GDP, while those labeled with a "H" correspond to those of the heptose moiety. α-ketoglutarate (α-KG) is a reaction byproduct. Additional details are provided in the text.

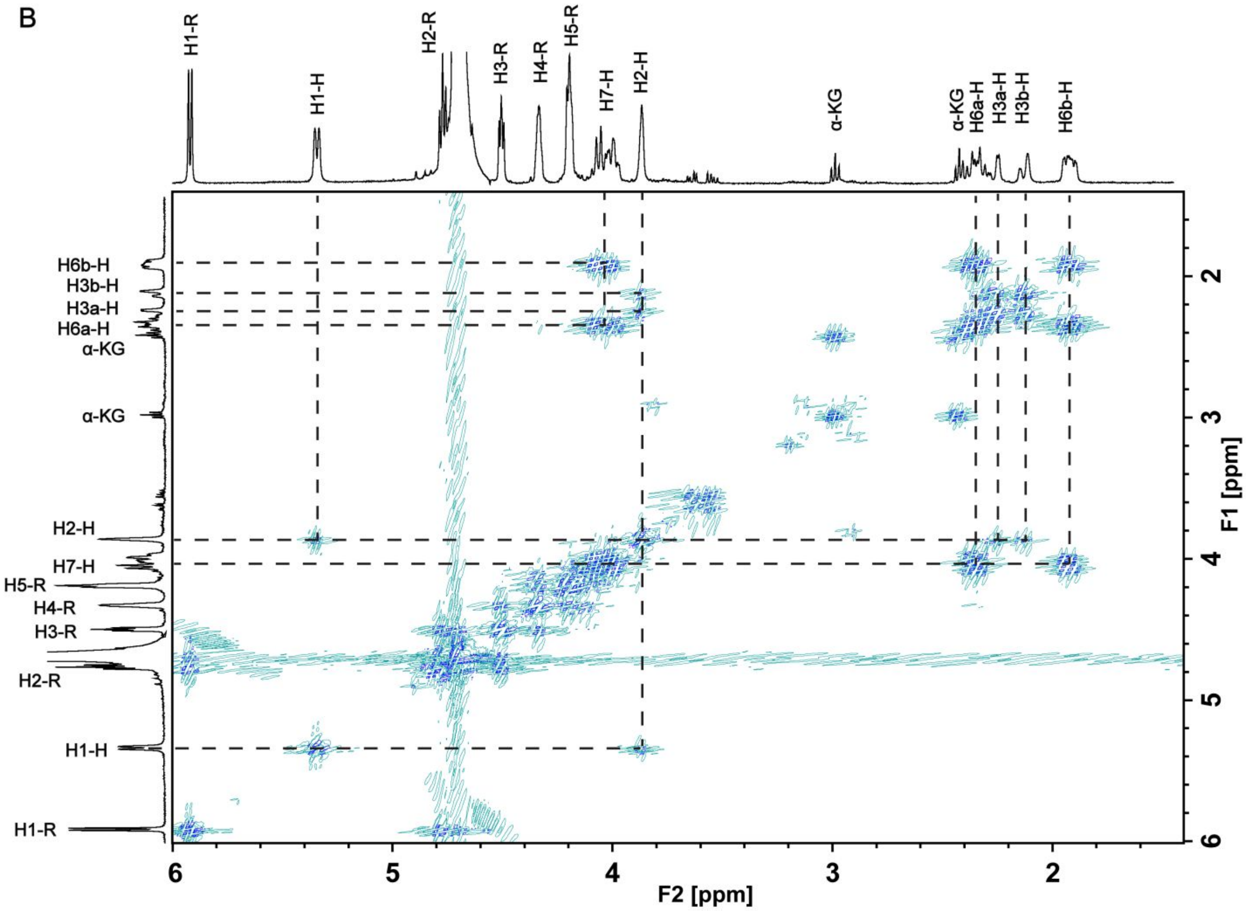

Figure S3. ^{1}H-^{1}H COSY NMR spectrum of GDP-3,6-dideoxy-4-keto-α-D-*threo*-heptose (**8**) prepared in D_2O using the C3-dehydratase from serotype HS:5. Resonances for the hydrogens labeled with an ''R'' correspond to the ribose moiety of GDP, while those labeled with a "H" correspond to those of the heptose moiety. α-Ketoglutarate (α-KG) is a reaction byproduct. Additional details are provided in the text

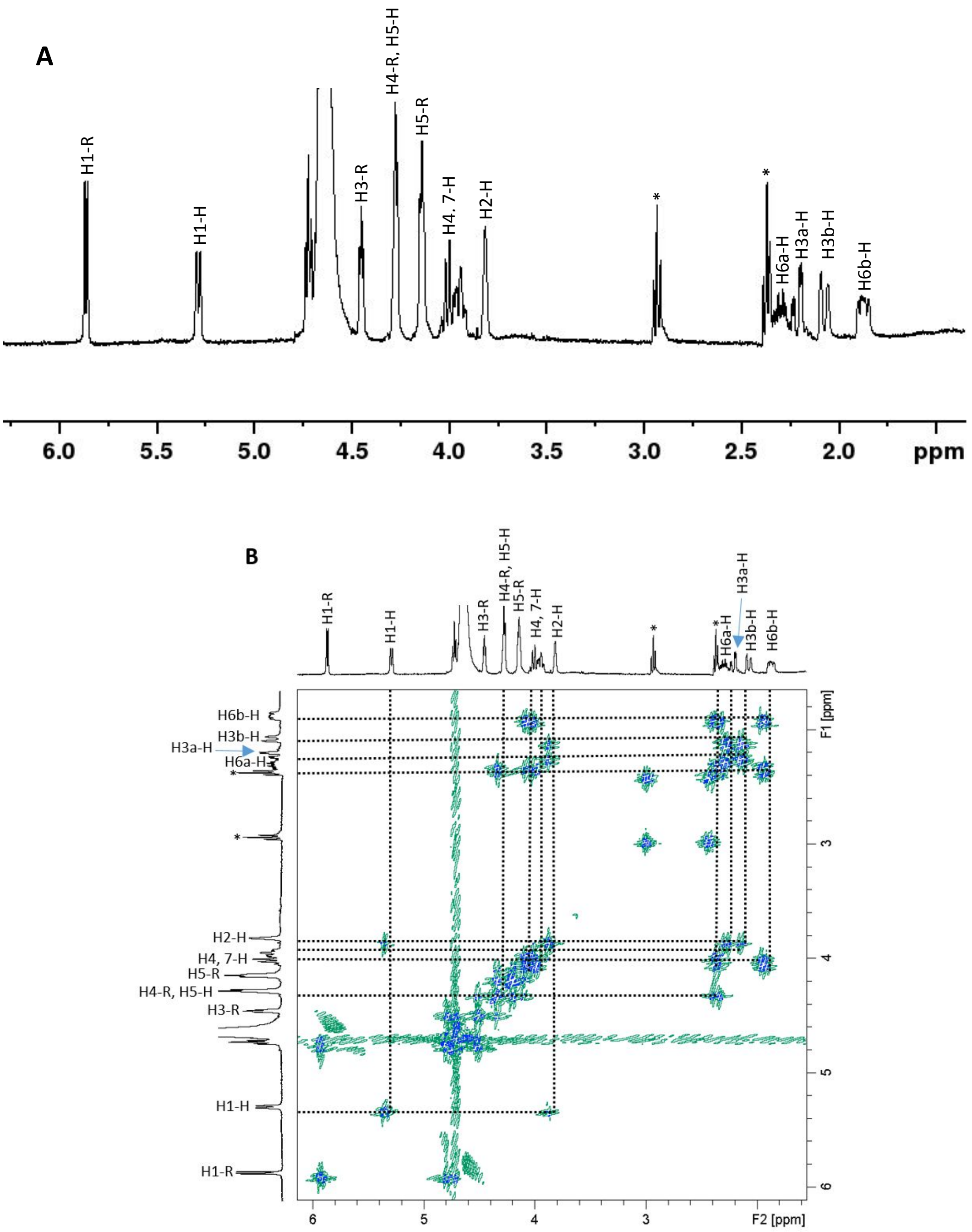

Figure S4. ^{1}H NMR and ^{1}H-^{1}H COSY NMR spectra of GDP-3,6-dideoxy-4-keto-α-D-*threo*-heptose (**8**) produced from the catalytic activity of the C3-dehydratase from serotype HS:11. Additional details are provided in the text. The reaction was conducted in H_2O. (A) ^{1}H NMR spectra and (B) ^{1}H-^{1}H COSY NMR spectra of GDP-3,6-dideoxy-4-keto-α-D-*threo*-heptose (**8**). Resonances for the hydrogens labeled with an ''R'' correspond to the ribose moiety and those labeled with a "H" correspond to those of the heptose moiety. The "*" indicates the resonances for α-keto-glutarate.

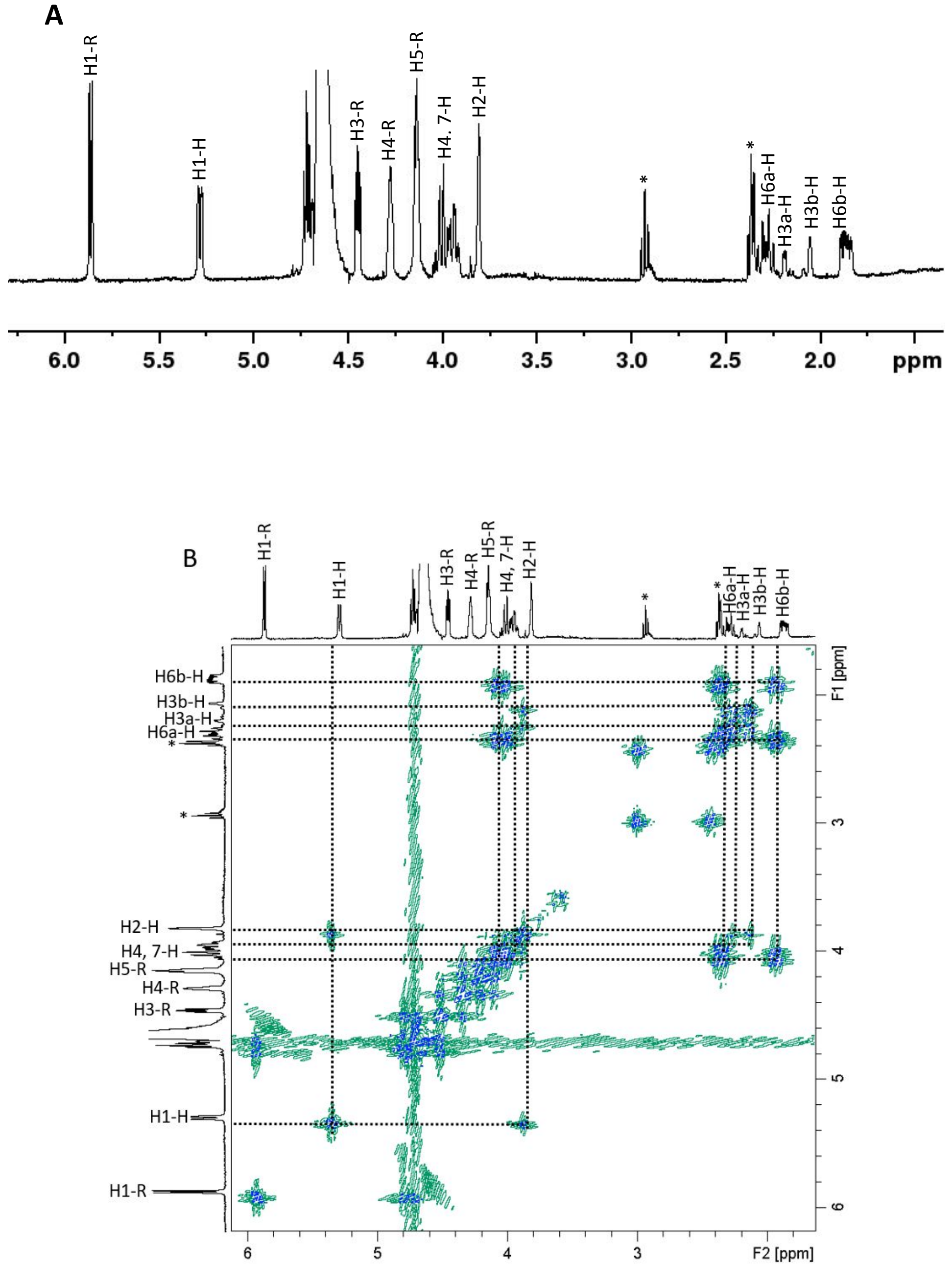

Figure S5. ^{1}H NMR and ^{1}H-^{1}H COSY NMR spectra of GDP-3,6-dideoxy-4-keto-α-D-*threo*-heptose (**8**) produced from the catalytic activity of the C3-dehydratase from serotype HS:11. Additional details are provided in the text. The reaction was conducted in D_2O. (A) ^{1}H NMR spectra and (B) ^{1}H-^{1}H COSY NMR spectra of GDP-3,6-dideoxy-4-keto-α-D-*threo*-heptose (**8**). Resonances for the hydrogens labeled with an ''R'' correspond to the ribose moiety and those labeled with a "H" correspond to those of the heptose moiety. The "*" indicates the resonances for α-keto-glutarate.

```
HS:2      1 MAIEFDIQESKILKGVYIITPNKFRDLRGEIWTAFTDEYLSKLVPDGIKFKHDKFINSHF  60
HS:15     1 MEIKFNIEESKILNGVYIITPNKFSDLRGDIWTAFTDEYLSNLVPNGIKFKHDKFINSHF  60
HS:32     1 MEIKFNIEESKILNGVYIITPNKFSDLRGDIWTAFTDEYLSNLVPNGIKFKHDKFINSHF  60
HS:42     1 MAIKFNIKESKILNGVYIITPNKFSDLRGDIWTAFTDEYLSNLVPNGIKFKHDKFINSHF  60
HS:63     1 ---------------MYIITPNKFRDLRGEIWTAFTDEHLSKLVPDGIKFKHDKFINSYF  45
-------------------------------------------------------------------------
HS:3      1 MAIEFDIQESKILKGVYIITPNKFRDLRGEIWTAFTSEAVDKLLPNGLKFIHDKFIHSKH  60
HS:4      1 MAIEFDIQESKILKGVYIITPNKFRDLRGEIWTAFTSEAVDKLLPNGLKFIHDKFIHSKH  60
HS:8      1 MAIKFDIQESKILKGVYIITPNKFRDLRGEIWTAFTSKAVDKLLPNGLKFIHDKFIHSKH  60
HS:10     1 MAIEFDIQESKILKGVYIITPNKFRDLRGEIWTAFTSEAIDNLLPNGLKFIHDKFIHSKH  60
HS:12     1 MAIEFDIQESKILKGVYIITPNKFRDLRGEIWTAFTSEAIDNLLPNGLKFIHDKFIHSKH  60
HS:23     1 MAIEFNIQESKILKGVYIITPNKFRDLRGEIWTAFTSKAVDKLLPNGLKFIHDKFIHSKH  60
HS:29     1 -MIEFDIQESKILKGVYIITPNKFRDLRGEIWTAFTSEAIDKLLPNGLKFIHDKFIHSKH  59
HS:33     1 MAIEFNIQESKILKGVYIITPNKFRDLRGEIWTAFISETIDKLLPNGLKFIHDKFIHSKH  60
HS:41     1 MAIEFDIQESKILKGVYIITPNKFRDLRGEIWTAFTDEAINKLLPSGLKFIHDKFIHSKH  60
HS:52     1 MAIEFDIQESKILKGVYIITPNKFRDLRGEIWTAFTSEAIDNLLPNGLKFIHDKFIHSKH  60
-------------------------------------------------------------------------
HS:5      1 MAIEFDIQESKILKGVYIITPNKFRDLRGEIWSAFSEDCLKHLIPDNLNFVLDKFTLSKP  60
HS:11     1 MAIEFDIQESKILKGVFIITPNSFKDLRGEIWSVFSSKDIESLLPSNLKFVLDKFTLSKH  60
HS:45     1 MAIEFDIQESKILKGVYIITPNKFRDLRGEIWSVFSEDCLKHLLPDNLNFVLDKFTLSKP  60


HS:2     61 NVLRGIHGDVKTYKLVTCVYGEVHQVVVDCRKDSPTYLKWEKFIISYKNQQLILLPPNMG 120
HS:15    61 NVLRGIHGDVKTYKLVTCVYGEVHQVVVDCRKDSPTYLKWEKFIISPRNQQLILLPPNMG 120
HS:32    61 NVLRGIHGDVKTYKLVTCVYGEVHQVVVDCRKNSPTYLKWEKFIISPRNQQLILLPPNMG 120
HS:42    61 NVLRGIHGDVKTYKLVTCVYGEVHQVVVDCRKDSPTYLKWEKFIISPRNQQLILLPPNMG 120
HS:63    46 NVLRGIHGDVKTYKLAACVYGEVHQVVVDCRKDSSTYLKWEKFIISSKNQQLILLPPNMG 105
-------------------------------------------------------------------------
HS:3     61 NVIRGIHGDVKTYKLATCVYGEVHQVVVDCRKDSPTYLKHERFIINQDNQKIILVPAGFG 120
HS:4     61 NVIRGIHGDVKTYKLATCVYGEVHQVVVDCRKDSPTYLKHERFIINQDNQKIILVPAGFG 120
HS:8     61 NVIRGIHGDVKTYKLATCVYGEVHQVVVDCRKDSPTYLKHERFIINQDNQQIILVPAGFG 120
HS:10    61 NVIRGIHGDVKTYKLATCVYGEIHQVVVDCRKDSPTYLKYEKFIINQDNQQIILVPAGFG 120
HS:12    61 NVIRGIHGDVKTYKLATCVYGEVHQVVVDCRKDSSTYLKHERFIINQDNQQIILVPAGFG 120
HS:23    61 NVIRGIHGDVKTYKLATCVYGEIHQVVVDCRKDSPTYLKYEKFIINQDNQQIILVPAGFG 120
HS:29    60 NVIRGIHGDVKTYKLATCVYGEIHQVVVDCRKDSPTYLKYEKFIINQDNQQIILVPAGFG 119
HS:33    61 NVIRGIHGDVKTYKLATCVYGEIHQVVVDCRKDSPTYLKHEKFIINQDNQQIILVPAGFG 120
HS:41    61 NVIRGIHGDVKTYKLATCVYGEIHQVVVDCRKDSPTYLKHERFIINQDNQQIILVPAGFG 120
HS:52    61 NVIRGIHGDVKTYKLATCVYGEVHQVVVDCRKDSPTYLKHERFIINQDNQQIILVPAGFG 120
-------------------------------------------------------------------------
HS:5     61 NVLRGIHGDHKSWKLVTCVYGEVHQVVVDCRKDSPTYLKWEKFIINQNNQKLILIPPYFG 120
HS:11    61 NVLRGIHGDHKSWKLVTCVYGEVHQVVVDCRKDSPTYLKWERFIINENNRKLILIPPYFG 120
HS:45    61 NVLRGIHGDHKSWKLVTCVYGEVHQVVVDCRKDSPTYLKWEKFIINQNNQKLILIPPYFG 120
```

```
HS:2    121 NSHYVSSK-EAVYYYKLAYEGEYMDAPDQFTYAWNDERIGIDWPTNTPILSDRDILATKN 179
HS:15   121 NSHYVSSK-EAVYYYKLAYEGEYLDAPDQFTYAWNDERIAIDWPTNSPILSERDILAMNK 179
HS:32   121 NSHYVSSK-EAVYYYKLAYEGEYLDAPDQFTYAWNDERIAIDWPTKSPILSERDILAMNK 179
HS:42   121 NSHYVSSK-EAVYYYKLAYKGEYLDAPDQFTYAWNDKRIAIDWPTNSPILSERDILAMNK 179
HS:63   106 NSHYVSSR-EAVYYYKLAYEGEYMDAPLINLLMHGMIVELL------------------- 145
-----------------------------------------------------------------------------
HS:3    121 NAHYVSSE-TAVYYYKCAYLGEYMDAPDQFTYAWNDERIGIDWPTNNPILSERDILAMSK 179
HS:4    121 NAHYVSSE-TAVYYYKCAYLGEYMDAPDQFTYAWNDERIGIDWPTNSPILSERDILAMSR 179
HS:8    121 NAHYVSSE-SAVYYYKCAYLGEYMDAHDQFTYAWNDERIGIDWPTNSPILSERDILATKN 179
HS:10   121 NAHYVSSE-SAVYYYKCAYKGDYADAPDQFTYAWNDERIGIDWPTNSPILSERDILAMSK 179
HS:12   121 NAHYVSSE-SAVYYYKCAYLGEYMDAHDQFTYAWNDERIGIDWPTNSPILSERDILAMSK 179
HS:23   121 NAHYVTSE-SAVYYYKCAYKGDYVDAPDQFTYAWNDERIGIDWPTNSPILSERDILATKN 179
HS:29   120 NAHYISSE-SAVYYYKCAYKGDYADAPDQFTYAWNDERIAIDWPTNSPILSERDILAMIK 178
HS:33   121 NAHYVSSE-SAVYYYKCAYLGEYMDAHDQFTYAWNDERIGIDWPTNSPILSERDILAMGK 179
HS:41   121 NAHYVSSE-SAVYYYKCAYKGDYADAPDQFTYAWNDERIGIDWPTNSPILSERDILAMSK 179
HS:52   121 NAHYVSSE-SAVYYYKCAYLGEYMDAHDQFTYAWNDERIGIDWPTNSPILSERDILAMSK 179
----------------------------------------------------------------------------
HS:5    121 NAQYVSSKSDALYYYKWAYEGEYVDAKEQFTYAWNDSRIAIDWPTNNPILSDRDIDATIN 180
HS:11   121 NAQYVSSKNDALYYYKWAYEGEYVDAKDQFTYAWNDSRIAIDWSTDTPILSDRDIDATIN 180
HS:45   121 NAQYVSSKSDALYYYKWAYEGEYVDAKDQFTYAWNDSRIAIDWATSNPILSDRDIDATIN 180


HS:2    180 KG---- 181
HS:15   180 DK---- 181
HS:32   180 DK---- 181
HS:42   180 DK---- 181
HS:63       ------ 145
----------------------
HS:3    180 DK---- 181
HS:4    180 DK---- 181
HS:8    180 KG---- 181
HS:10   180 DK---- 181
HS:12   180 DK---- 181
HS:23   179 KG---- 181
HS:29   180 DK---- 180
HS:33   180 DK---- 181
HS:41   180 DK---- 181
HS:52   180 DK---- 181
----------------------
HS:5    181 DHTKGF 186
HS:11   181 DHIKG- 185
HS:45   181 DHTKGF 186
```

Figure S6: Multiple sequence alignment for the epimerases from serotypes HS:2, HS:3, HS:4, HS:5, HS:8, HS:10, HS:11, HS:12, HS:15, HS:23, HS:29, HS:32, HS;33, HS:41, HS:42, HS:45, HS:52, and HS:63. The residues conserved in the C3-epimerases, C3/C5-epimerases, and C5-epimerases are highlighted in yellow, gray, and green, respectively. Two active site general acid/base groups (histidine and tyrosine) are highlighted in pink. Those residues that are fully conserved in all of the enzymes are highlighted in blue.

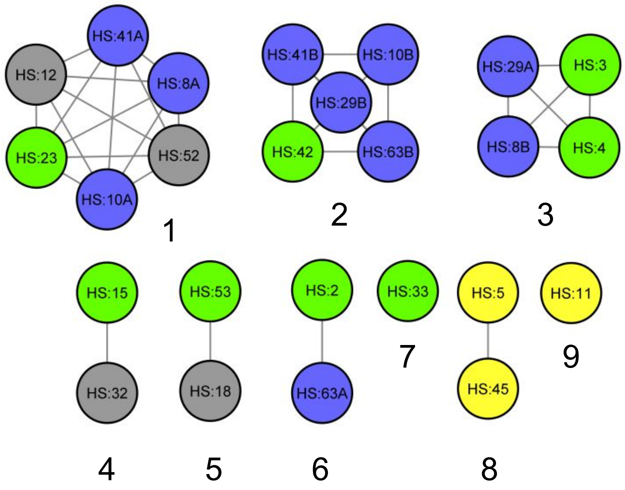

Figure S7: Sequence similarity network for 25 C4-reductases identified within various strains of *C. jejuni* at a sequence identity cutoff of 89%. The green and blue colored nodes denote C4-reductases that have previously been isolated and the products characterized. The yellow-colored nodes indicate C4-reductases involved in the biosynthesis of 3,6-dideoxy heptoses that are characterized in this investigation. The grey-colored nodes are additional C4-reductases that have not been purified. The product specificities for the numbered clusters are as follows: Group-1 (GDP-6-deoxy-D-*altro*-heptose (**4**)); Group-2 (GDP-6-deoxy-L-*galacto*-heptose (**6**)); Group-3 (GDP-6-deoxy-D-*ido*-heptose (**5**)); Group-4 (GDP-6-deoxy-L-*gulo*-heptose (**8**)); Group-5 (GDP-6-deoxy-D-*manno*-heptose (**3**)); Group-6 (GDP-6-deoxy-L-*gluco*-heptose (**7**)); Group-7 (GDP-6-deoxy-D-*ido*-heptose (**5**)); Group-8 (GDP-6-deoxy-L-*ribo*-heptose); and Group-9 (GDP-6-deoxy-L-*xylo*-heptose).

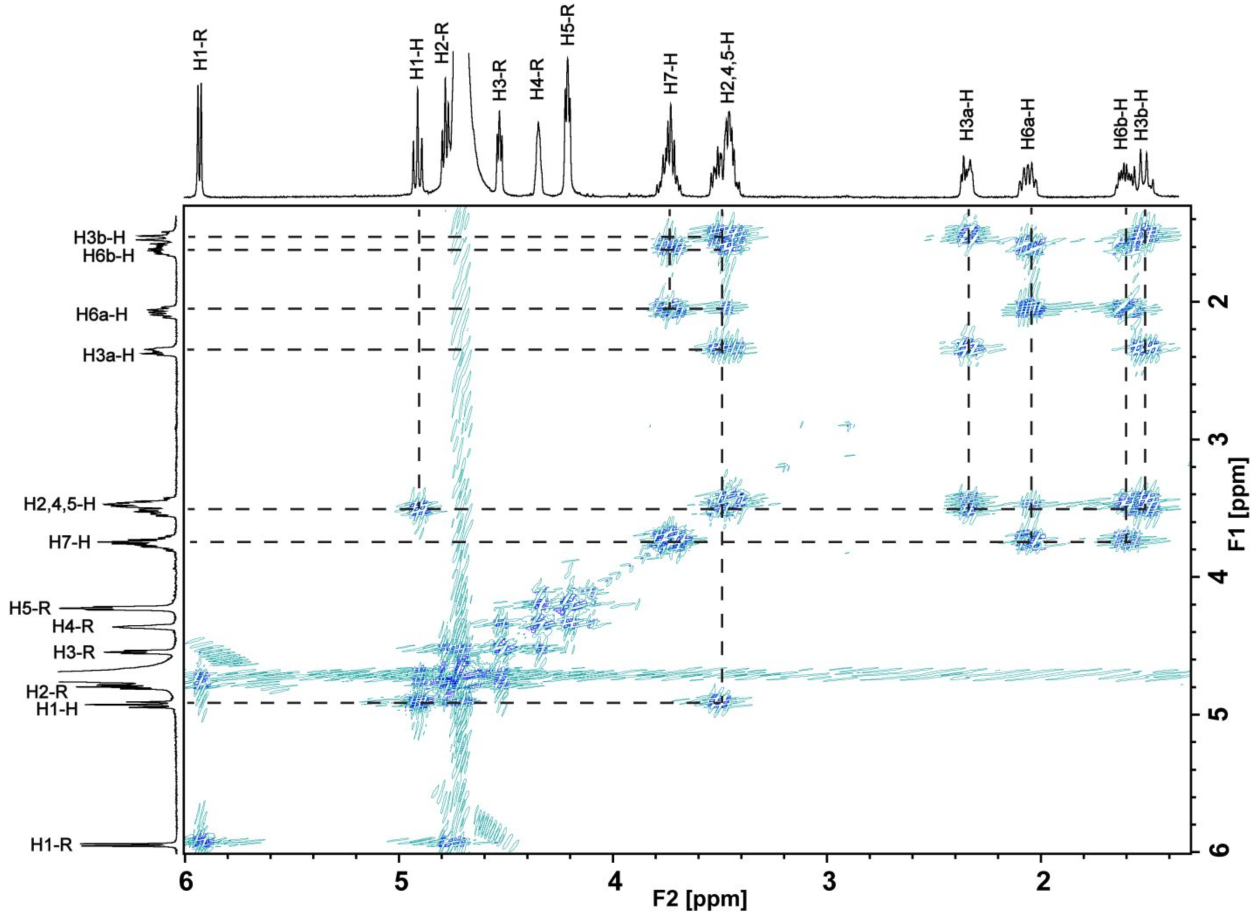

Figure S8. ^1H-^1H NMR spectra of GDP-3,6-dideoxy-β-L-*ribo*-heptose (**12**) using the C4-reductase from serotype HS:5. Reaction conducted in H_2O. Resonances for the hydrogens labeled with an ''R'' correspond to the ribose moiety of GDP, while those labeled with a "H" correspond to those of the heptose moiety. Additional details are provided in the text.

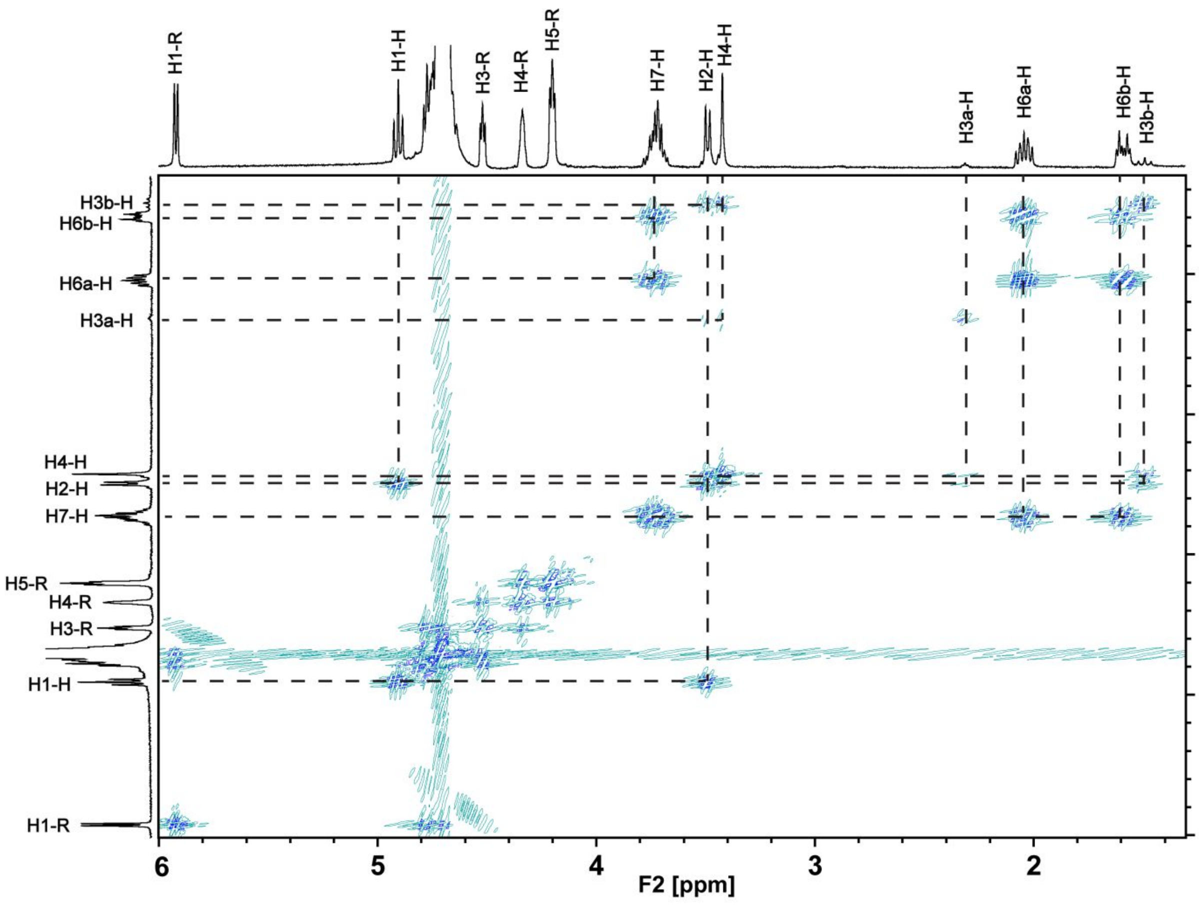

Figure S9. ^{1}H-^{1}H NMR spectra of GDP-3,6-dideoxy-β-L-*ribo*-heptose (**12**) using the C4-reductase from serotype HS:5. Reaction conducted in D_2O. Resonances for the hydrogens labeled with an ''R'' correspond to the ribose moiety of GDP, while those labeled with a "H" correspond to those of the heptose moiety. Additional details are provided in the text.

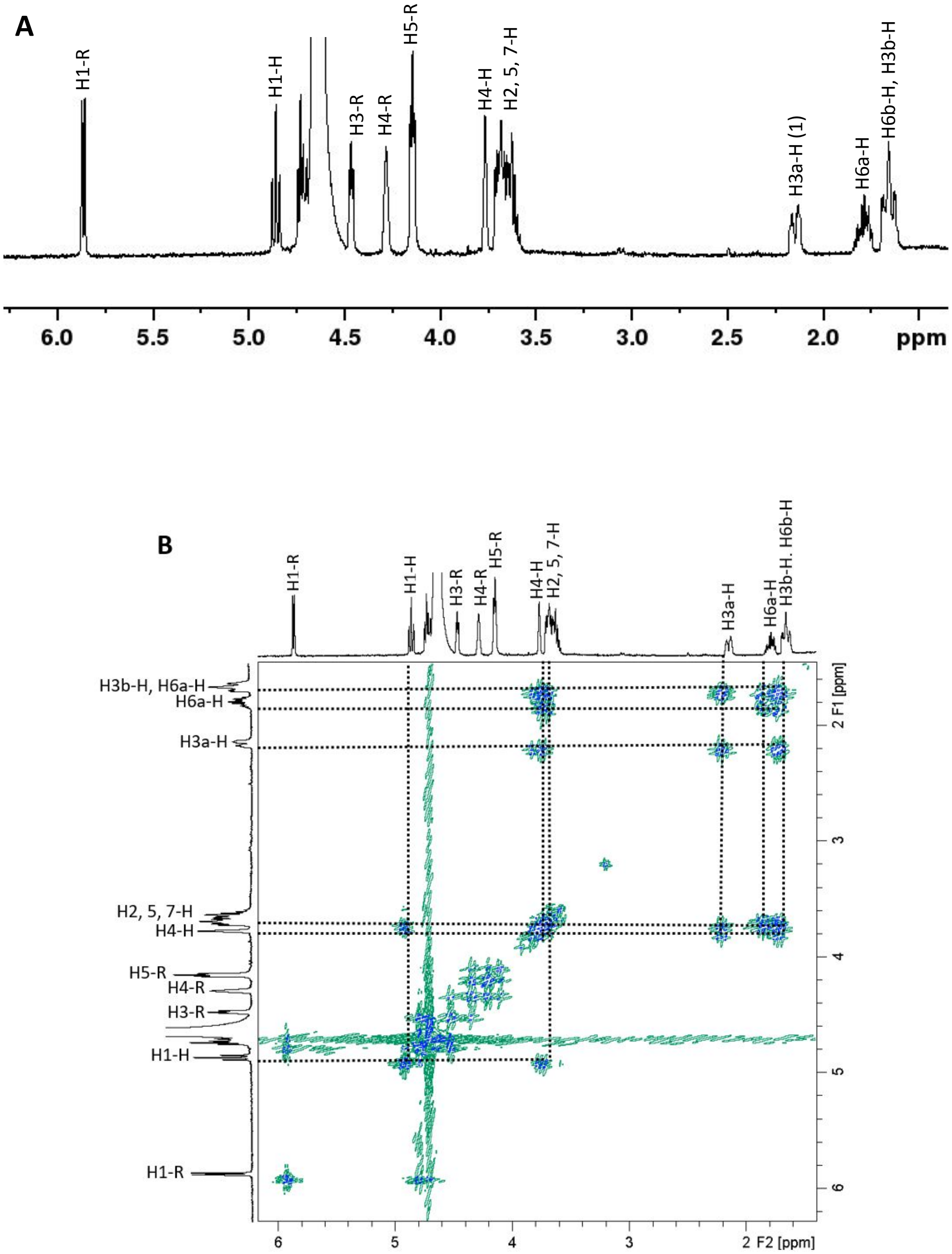

Figure S10. ^{1}H NMR and ^{1}H-^{1}H COSY NMR spectra of GDP-3,6-dideoxy-β-L-*xylo*-heptose (**13**) produced from the catalytic activity of the C4-reductase from serotype HS:11. Additional details are provided in the text. The reaction was conducted in H_2O. (A) ^{1}H NMR spectra and (B) ^{1}H-^{1}H COSY NMR spectra of GDP-3,6-dideoxy-β-L-*xylo*-heptose (**13**). Resonances for the hydrogens labeled with an ''R'' correspond to the ribose moiety and those labeled with a "H" correspond to those of the heptose moiety.

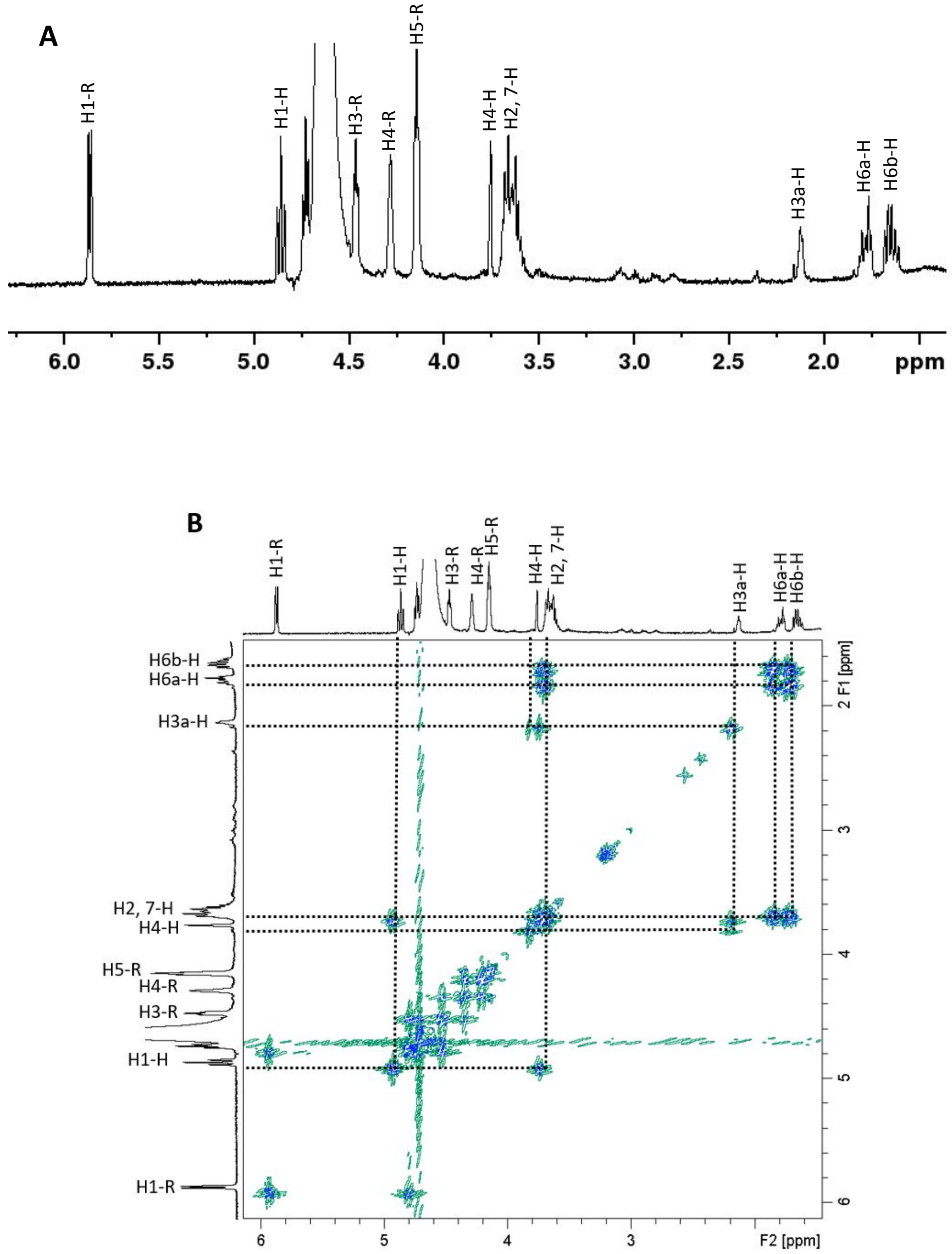

Figure S11. ^{1}H NMR and ^{1}H-^{1}H COSY NMR spectra of GDP-3,6-dideoxy-β-L-*xylo*-heptose (**13**) produced from the catalytic activity of the C4-reductase from serotype HS:11. Additional details are provided in the text. The reaction was conducted in D_2O. (A) ^{1}H NMR spectra and (B) ^{1}H-^{1}H COSY NMR spectra of GDP-3,6-dideoxy-β-L-*xylo*-heptose (**13**). Resonances for the hydrogens labeled with an ''R'' correspond to the ribose moiety and those labeled with a "H" correspond to those of the heptose moiety.

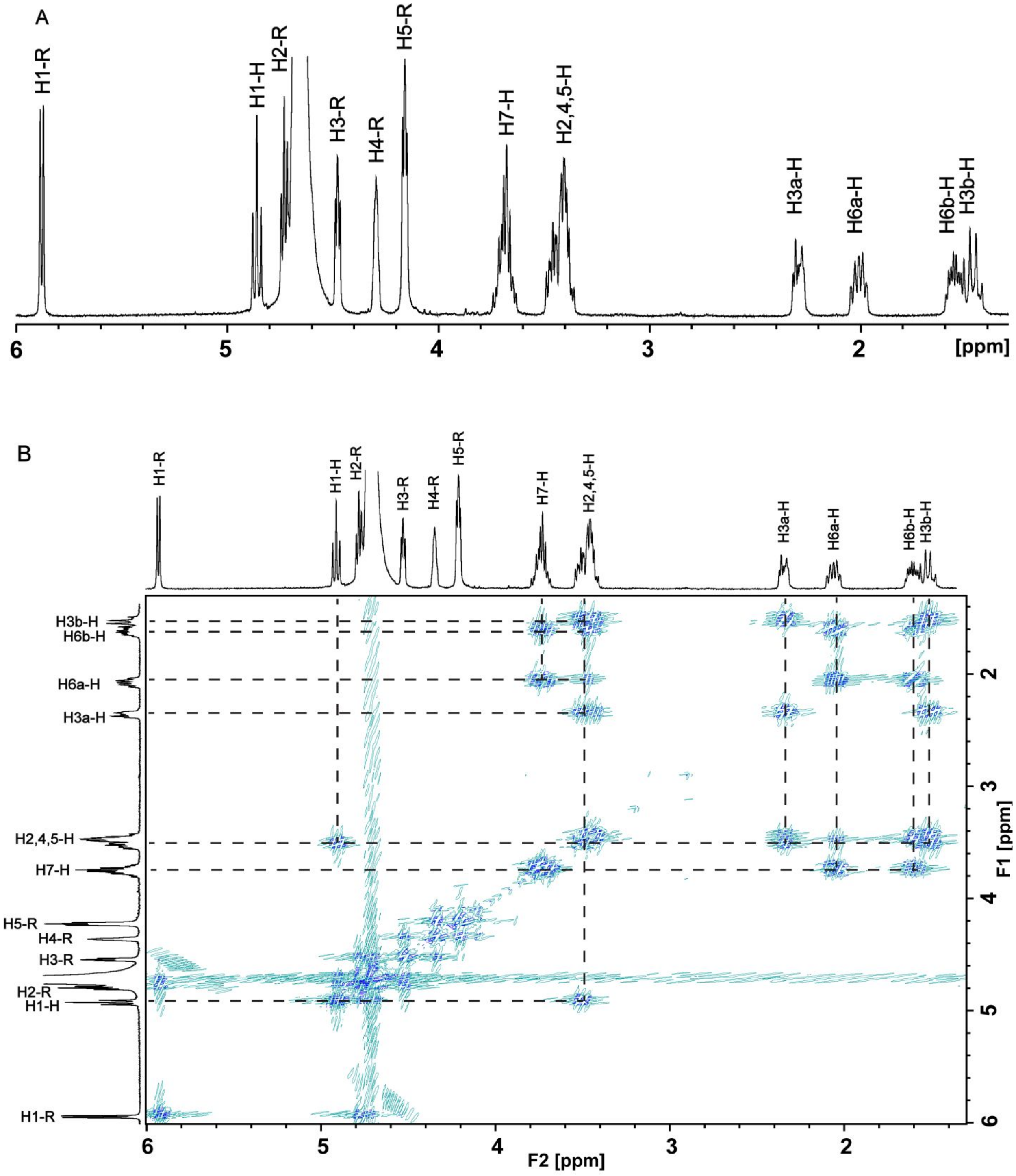

Figure S12. ^{1}H NMR and ^{1}H-^{1}H COSY NMR spectra of GDP-3,6-dideoxy-β-L-*ribo*-heptose (**12**) produced from the catalytic activity of the C4-reductase from serotype HS:2. Additional details are provided in the text. The reaction was conducted in H_2O. (A) ^{1}H NMR spectra and (B) ^{1}H-^{1}H COSY NMR spectra of GDP-3,6-dideoxy-β-L-*ribo*-heptose (**12**). Resonances for the hydrogens labeled with an ''R'' correspond to the ribose moiety and those labeled with a "H" correspond to those of the heptose moiety.

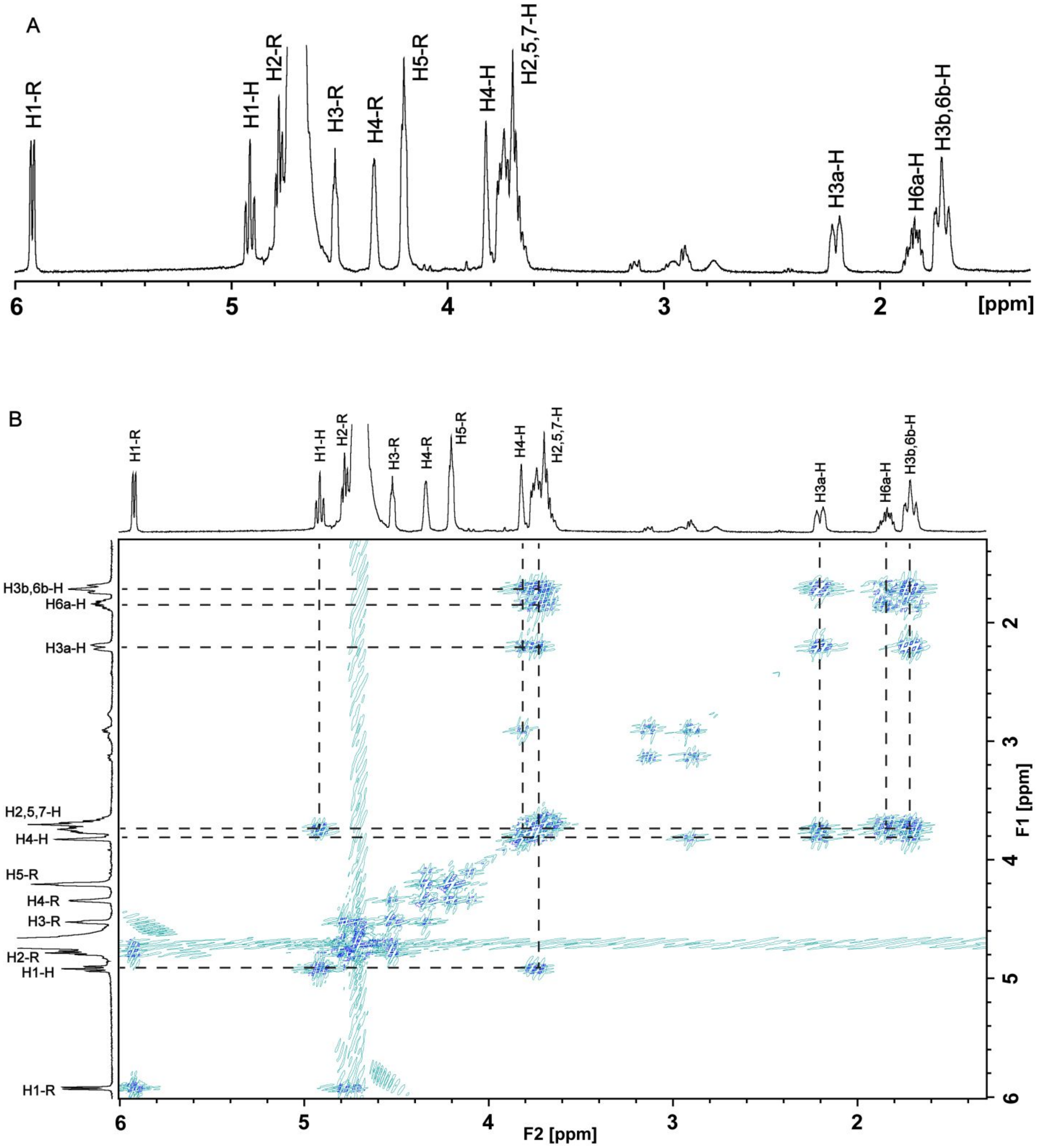

Figure S13. ^1H NMR and ^1H-^1H COSY NMR spectra of GDP-3,6-dideoxy-β-L-*xylo*-heptose (**13**) produced from the catalytic activity of the C4-reductase from serotype HS:15. Additional details are provided in the text. The reaction was conducted in H_2O. (A) ^1H NMR spectra and (B) ^1H-^1H COSY NMR spectra of GDP-3,6-dideoxy-β-L-*xylo*-heptose (**13**). Resonances for the hydrogens labeled with an ''R'' correspond to the ribose moiety and those labeled with a "H" correspond to those of the heptose moiety.

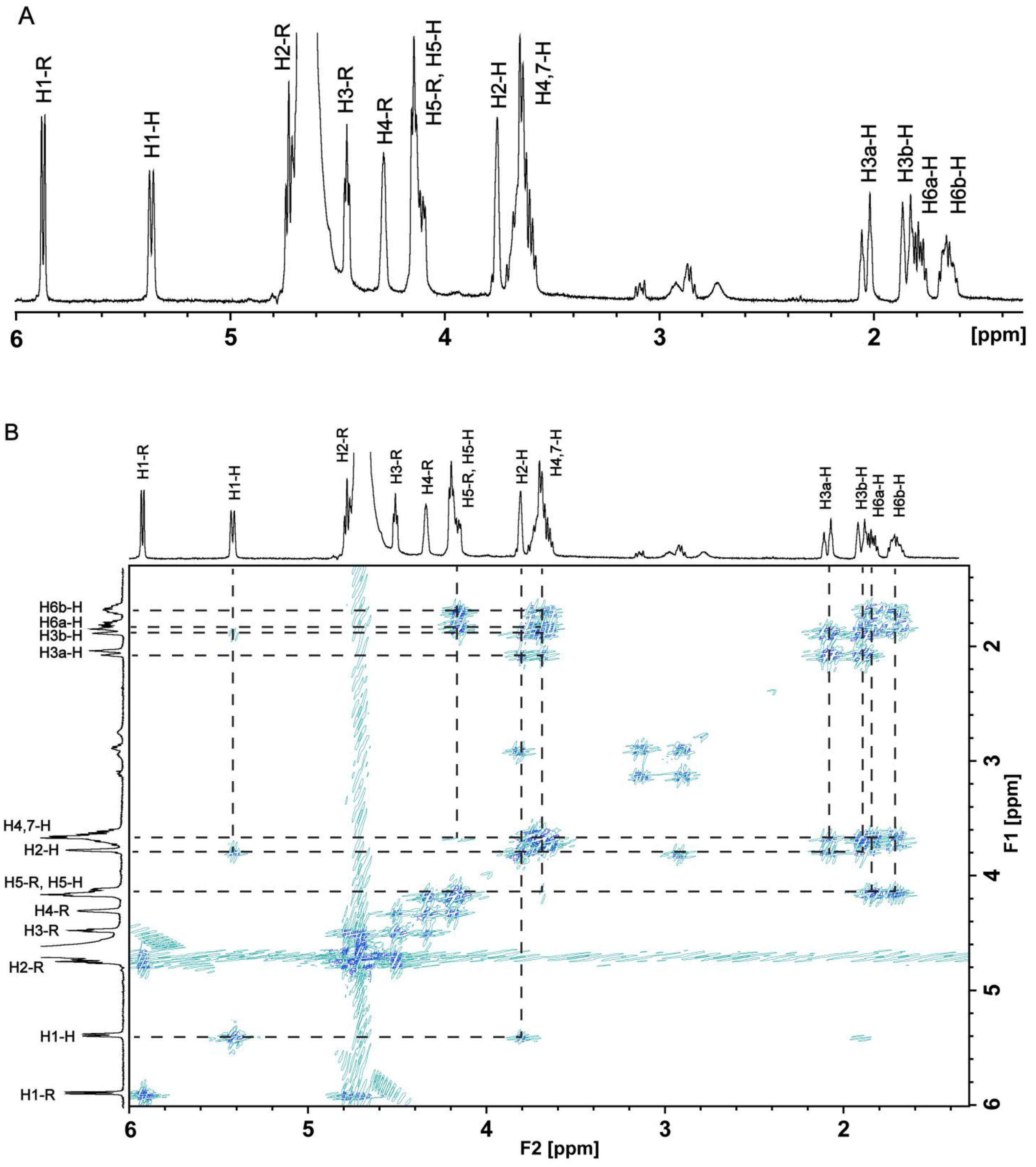

Figure S14. ^{1}H NMR and ^{1}H-^{1}H COSY NMR spectra of GDP-3,6-dideoxy-α-D-*lyxo*-heptose (**15**) produced from the catalytic activity of the C4-reductase from serotype HS:3. Additional details are provided in the text. The reaction was conducted in H_2O. (A) ^{1}H NMR spectra and (B) ^{1}H-^{1}H COSY NMR spectra of GDP-3,6-dideoxy-α-D-*lyxo*-heptose (**15**). Resonances for the hydrogens labeled with an ''R'' correspond to the ribose moiety and those labeled with a "H" correspond to those of the heptose moiety.

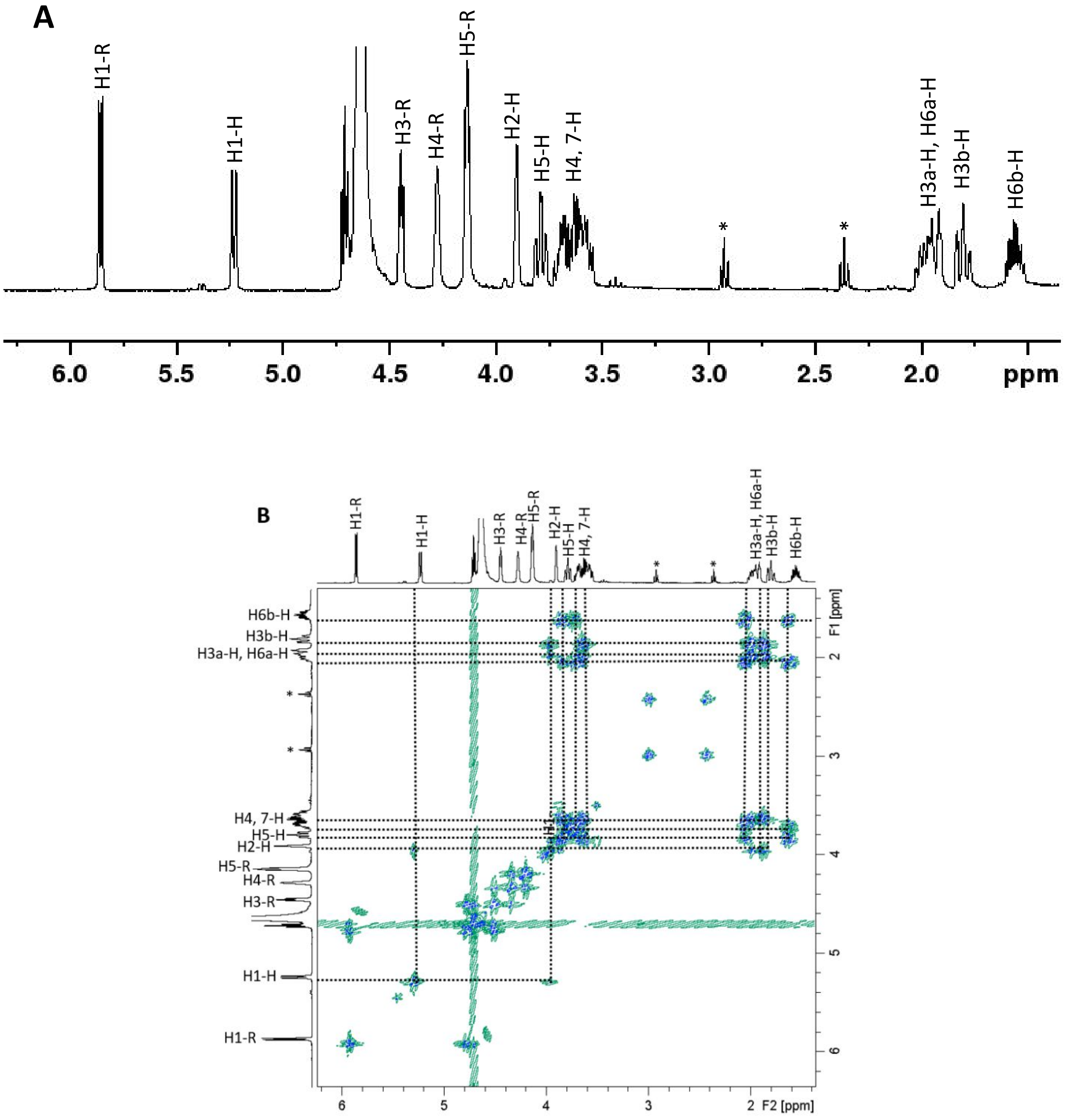

Figure S15. ^{1}H NMR and ^{1}H-^{1}H COSY NMR spectra of GDP-3,6-dideoxy-α-D-*arabino*-heptose (**14**) produced from the catalytic activity of the C4-reductase from serotype HS:53. Additional details are provided in the text. The reaction was conducted in H_2O. (A) ^{1}H NMR spectra and (B) ^{1}H-^{1}H COSY NMR spectra of GDP-3,6-dideoxy-α-D-*arabino*-heptose (**14**). Resonances for the hydrogens labeled with an ''R'' correspond to the ribose moiety and those labeled with a "H" correspond to those of the heptose moiety. The "*" indicates the resonances for α-keto-glutarate.
